# Structural basis for lipid and copper regulation of the ABC transporter MsbA

**DOI:** 10.1038/s41467-022-34905-2

**Published:** 2022-11-26

**Authors:** Jixing Lyu, Chang Liu, Tianqi Zhang, Samantha Schrecke, Nicklaus P. Elam, Charles Packianathan, Georg K. A. Hochberg, David Russell, Minglei Zhao, Arthur Laganowsky

**Affiliations:** 1 Department of Chemistry, Texas A&M University, College Station, 77843 TX USA; 2 Department of Biochemistry and Molecular biology, University of Chicago, Chicago, 60637 IL USA; 3 Max Planck Institute for Terrestrial Microbiology and Department of Chemistry, University of Marburg, Marburg, Germany; 4 Center for Synthetic Microbiology (SYNMIKRO), Department of Chemistry, University of Marburg, Marburg, Germany; 5 Present Address: Walter Reed Army Institute of Research, Pilot Bioproduction Facility, Silver Spring, 20910 MD USA

**Keywords:** Cryoelectron microscopy, Membrane proteins, Bacterial structural biology, Mass spectrometry

## Abstract

A critical step in lipopolysaccharide (LPS) biogenesis involves flipping lipooligosaccharide, an LPS precursor, from the cytoplasmic to the periplasmic leaflet of the inner membrane, an operation carried out by the ATP-binding cassette transporter MsbA. Although LPS binding to the inner cavity of MsbA is well established, the selectivity of MsbA-lipid interactions at other site(s) remains poorly understood. Here we use native mass spectrometry (MS) to characterize MsbA-lipid interactions and guide structural studies. We show the transporter co-purifies with copper(II) and metal binding modulates protein-lipid interactions. A 2.15 Å resolution structure of an N-terminal region of MsbA in complex with copper(II) is presented, revealing a structure reminiscent of the GHK peptide, a high-affinity copper(II) chelator. Our results demonstrate conformation-dependent lipid binding affinities, particularly for the LPS-precursor, 3-deoxy-D-*manno*-oct-2-ulosonic acid (Kdo)_2_-lipid A (KDL). We report a 3.6 Å-resolution structure of MsbA trapped in an open, outward-facing conformation with adenosine 5’-diphosphate and vanadate, revealing a distinct KDL binding site, wherein the lipid forms extensive interactions with the transporter. Additional studies provide evidence that the exterior KDL binding site is conserved and a positive allosteric modulator of ATPase activity, serving as a feedforward activation mechanism to couple transporter activity with LPS biosynthesis.

## Abstract (web summary)

The bacterial ABC transporter MsbA is essential for lipopolysaccharide biogenesis. Here, the authors apply native mass spectrometry, X-ray crystallography, cryo-EM and biochemical approaches to characterize the structural basis and functional roles of MsbA binding to copper and specific lipids.

## Introduction

**Fig. 1:**
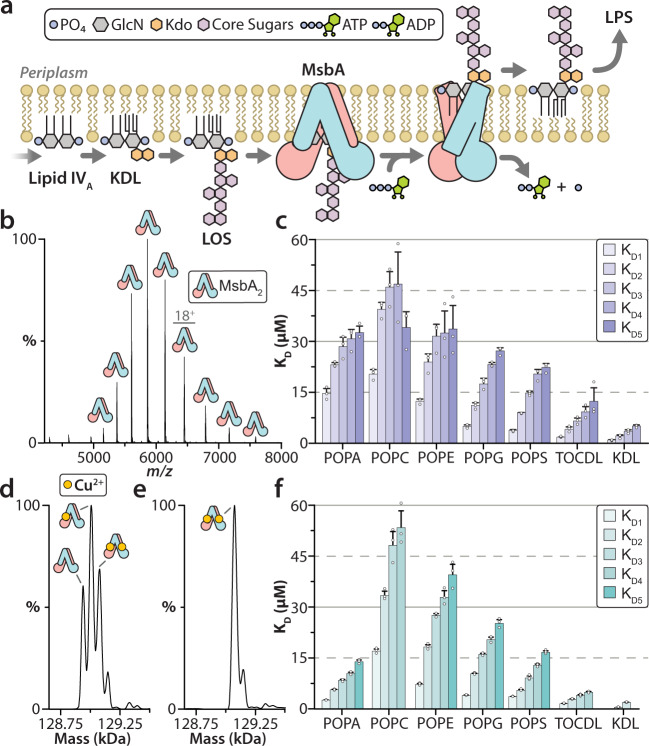
Copper(II) binding to MsbA modulates lipid binding affinity. **a** Lipopolysaccharide biosynthesis commences in the cytoplasm to generate lipooligosaccharide (LOS). LOS is composed of conserved lipid A structure (gray), a bisphosphorylated disaccharide of glucosamine, modified with 3-deoxy-D-*manno*-oct-2-ulosonic acid (Kdo) sugar (orange) and core oligosaccharide (purple), of which is dependent on the bacteria. MsbA, powered by the hydrolysis of ATP, flips cytoplasmic LOS from inner to outer leaflet of the inner membrane, an essential step in LPS biogenesis. The flipped LOS is transported to the outer membrane along with additional modifications to become LPS. **b** Native mass spectrum of optimized MsbA samples in C_10_E_5_ yields a well-resolved mass spectrum. **c** Equilibrium dissociation constants (K_D_) for individual lipid binding events to partially loaded MsbA. **d** Deconvolution of the mass spectrum shown in panel **b**. The different molecular species correspond to dimeric MsbA and different numbers of bound copper ions. **e** Measured mass of MsbA after loading with copper(II) shows saturation of two binding sites. **f** K_D_s for individual lipid binding events to MsbA fully loaded with copper(II). Reported are the mean and standard deviation (*n* = 3, biological replicates). Source data are provided as a Source Data file.

A defining feature of most Gram-negative bacteria is the presence of lipopolysaccharide (LPS) in the outer leaflet of the outer membrane^1–3^. LPS contributes to the formation of an impermeable barrier that helps bacteria resist antibiotics and environmental stresses^2^. Biogenesis of LPS commences in the cytoplasm with the production of the LPS-precursor lipooligosaccharide (LOS) followed by an orchestrated transport to the cell surface along with further modifications (Fig. 1a)^4^. LOS contains a conserved lipid A moiety, a bisphosphorylated disaccharide of glucosamine (GlcN) with four to seven acyl chains, that is decorated with 3-deoxy-D-*manno*-oct-2-ulosonic acid (Kdo) sugar^1^. Further decoration includes the attachment of a core oligosaccharide, which varies in different bacteria^2^. Cytoplasmic LOS is flipped from the inner to the periplasmic leaflet of the inner membrane, an essential step carried out by the ATP-Binding Cassette (ABC) transporter MsbA. As inhibition or deletion of MsbA is lethal^5^, the transporter has emerged as an attractive target for developing antibiotics. Small molecule MsbA inhibitors have recently been developed that vary in mode of action, such as trapping the transporter in an inward-facing (IF) conformation or mimicking substrate binding^6–10^.

A number of studies employing an arsenal of biophysical techniques have provided mechanistic and structural insight into MsbA^6,11–20^. MsbA forms a homodimer, each subunit consists of a transmembrane domain (TMD, six transmembrane helices per subunit) and a cytosol-exposed nucleotide-binding domain (NBD)^21^. The proposed mechanism by which MsbA translocates LOS across the bilayer involves several steps^6,17^. Apo or adenosine 5’-diphosphate (ADP) bound MsbA populates an IF conformation with the NBDs separated in space, promoting entry and binding of the bulky LOS. The binding of LOS in the central, interior cavity and adenosine 5’-triphosphate (ATP) to MsbA promotes dimerization of the NBDs. ATP hydrolysis powers a conformational change to an outward-facing (OF) conformation, transporting LOS to the periplasmic side of the inner membrane. LOS and inorganic phosphate are released and MsbA cycles back to an IF conformation. Like many other ABC transporters, the ATPase activity of MsbA is stimulated in the presence of different substrates, particularly hexaacylated lipid A species, such as 3-deoxy-D-*manno*-oct-2-ulosonic acid (Kdo)_2_-lipid A (KDL)^22–24^. Although the highly selective recognition of LPS in the inner cavity by MsbA is well understood^6,17^, the structural basis of stimulation of MsbA ATPase activity for lipids binding to other site(s) remains poorly understood.

Native mass spectrometry (MS) has emerged as an indispensable biophysical technique to study membrane protein complexes and their interactions with lipids and other molecules^25^. With the ability to preserve non-covalent interactions and native-like structure of membrane proteins in the gas phase^26,27^, the technique has provided insightful information on various biochemical interactions including nucleotide, drug, peptide and lipid binding as well as yielding thermodynamic data for protein-protein, protein-ligand and protein-lipid interactions^28–35^. In this study, we set out to characterize MsbA-lipid interactions in different conformational states: apo, inward-facing; and vanadate-trapped, outward-facing. Native MS studies reveal MsbA co-purifies with copper(II) but also MsbA-lipid interactions are directly influenced by metal binding and protein conformation. Structural studies reveal a KDL binding site that has not been previously observed in other ABC structures. Our findings bring forth new insights into metal and lipid regulation of MsbA.

## Results and discussion

### Discovery of copper(II)-bound MsbA

As MsbA has been reported to co-purify with LPS and other lipids^17,36^, our first objective was to optimize the purification of MsbA from *E. coli* for native MS studies. The mass spectrum of MsbA purified in the detergent n-dodecyl-β-D-maltopyranoside (DDM) was a broad hump (Supplementary Figure 1), indicating a highly heterogenous sample, corresponding to a battery of co-purified small molecule contaminants. The sharp mass spectral peaks decorating the underlying hump, centered around 4500 *m/z*, correspond to monomeric MsbA, resulting from dissociation of the homodimer under the high activation, non-native conditions. After employing an established detergent screening method to optimize protein purification (see Supplementary Note 1)^37^, MsbA samples solubilized in the pentaethylene glycol monodecyl ether (C_10_E_5_) detergent exhibited a well-resolved mass spectrum (Fig. 1b). The MsbA samples also hydrolyzed ATP over time as evident by the presence ADP whereas MsbA containing the E506Q mutation, which abolishes catalytic activity^38^, did not turn over ATP (Supplementary Fig. 1c–d). Interestingly, different molecular species are measured, corresponding to dimeric MsbA and the addition of one to three ~65 Da adducts (Fig. 1d and Supplementary Table 1). Analysis of MsbA samples using inductively coupled plasma mass spectrometry (ICP-MS) identified the bound adducts as copper (Supplementary Tables 2–3). The addition of copper(II) to MsbA saturated the two binding sites (Fig. 1e). Removal of excess copper(II) nor the addition of the copper(II) chelator, trientine^39^ reduced the amount of metal bound to MsbA (Supplementary Fig. 2). These results reveal MsbA has one high-affinity copper(II) binding site per subunit.

### Determination of MsbA-lipid binding affinities

To better understand MsbA-lipid interactions, we determined equilibrium binding constants for MsbA binding to different lipids. For these studies, we selected 1,1′,2,2′-tetraoleoyl-cardiolipin (TOCDL, 72:4) or phosphatidic acid (PA), phosphatidylcholine (PC), phosphatidylethanolamine (PE), phosphatidylglycerol (PG), and phosphatidylserine (PS) containing the acyl chain composition, 1-palmitoyl-2-oleoyl (PO, 16:0-18:1). We also included 3-deoxy-D-*manno*-oct-2-ulosonic acid (Kdo)_2_-lipid A (KDL), an LPS precursor known to stimulate MsbA ATPase activity^22–24^. Except for PC, these lipids are found in *E. coli*^40^. MsbA partially and fully loaded with copper(II) was titrated with each lipid followed by recording their native mass spectra (Supplementary Figs. 3–6). The mole fractions of apo and lipid bound states of MsbA were extracted from the deconvoluted MS data and used to determine the equilibrium dissociation constant (K_D*N*_) for the *N*th binding event (Supplementary Tables 4–5). MsbA loaded with copper(II) resulted in a statistically significant enhancement in binding affinities for POPA and POPS (Fig. 1c–f and Supplementary Fig. 7). The two tightest binding lipids for MsbA loaded with copper(II) were TOCDL (K_D1_ = 1.6 µM) and KDL (K_D1_ = 0.6 µM). POPG binding affinities were largely independent of the copper(II) bound state of MsbA. In short, these results demonstrate that MsbA not only binds selectively to lipids but is also dependent on the degree of copper(II) bound to the transporter.

### X-ray structure of the N-terminus of MsbA coordinated to copper

**Fig. 2:**
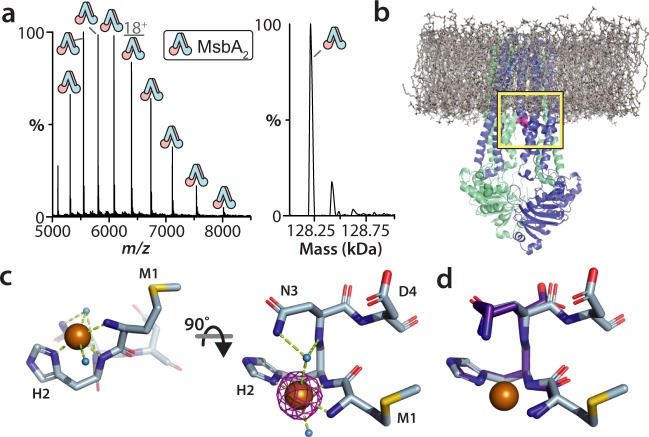
The N-terminus of MsbA binds copper(II) and its crystal structure. **a** Native mass spectrum and deconvolution of MsbA with deletion of four N-terminal residues. No copper(II) is bound to the truncated transporter. **b** Snapshot from a molecular dynamics simulation of MsbA in a 16:0 PC (DPPC) bilayer (PDB 6BPP downloaded from MemProtMD^44^). DPPC is shown in stick representation (grey). Protein is shown in cartoon representation with residues 4-8 colored pink. Yellow box highlights the location of the N-terminus relative to the inner membrane. **c** Structure of the N-terminus of MsbA (residues 1-4) fused to the green fluorescent protein (GFP) coordinating copper(II). The N-terminal peptide is shown in stick representation with water (blue) and copper(II) shown as spheres. Bonds are shown as dashed lines (limon). Anomalous difference peak shown in magenta and contoured at 15 sigma. The structure of GFP is omitted for clarity. **d** Alignment of the N-terminal MsbA peptide bound to copper(II) with GHK-copper(II) complex (CCDC-809108, Cα colored purple).

Two putative metal binding sites for a MsbA have been previously reported^41^. Mutation of one of the putative sites (H562A and H576A) did not abolish copper(II) binding (Supplementary Fig. 2). After carefully inspecting MsbA structures with focus on histidine and cysteine residues, both of which are known to preferentially coordinate copper^42^, we noted that in all MsbA structures the N-terminal histidine is not observed, which is likely due to a flexible linker or populating different structures. Removal of the first four residues (Met-His-Asn-Asp) of MsbA abolished binding of copper(II) (Fig. 2a), pinning down the metal binding site to the N-terminus. Additional studies show the truncated, copper(II)-free protein does not display altered ATPase activity in detergent nor proteoliposomes (Supplementary Figure 8). As the initiating Met has been shown to be removed for some bacterial proteins^43^, we expressed and purified MsbA with a C-terminal affinity tag and found it retains an intact N-terminus that can also bind copper(II) (Supplementary Figure 2). Interestingly, MD simulations^44^ show the N-terminus of MsbA in different structures are located near the inner membrane and in a region where LOS could pass before entering the interior cavity (Fig. 2b and Supplementary Figure 9).

To facilitate structure determination, the N-terminal sequence of MsbA was grafted onto proteins known to readily crystallize. Native MS shows these fusion proteins containing a fragment of the N-terminus of MsbA of variable length bound copper(II) and monomeric (Supplementary Fig. 10). One of the green fluorescent protein (GFP) fusions, containing residues 1-4 of MsbA, produced X-ray-grade crystals that led to structure determination at 2.15 Å resolution (Supplementary Table 6). Resolved electron density for the N-terminus is observed along with strong anomalous signal for the bound copper ion (Fig. 2c and Supplementary Fig. 11) and adopts a structure of similar to that for the copper(II) coordinated GHK peptide, a naturally occurring high-affinity copper(II) chelator found in the blood plasma (Fig. 2d)^45^. The copper(II) adopts a pseudo-octahedral coordination with the planar ligands comprised of the amine of M1, amide and sidechain of H2. An additional interaction is formed by the sidechain of D197’ from a symmetry related molecule (Supplementary Fig. 10c) and similar to the GHK-copper(II) structure, wherein is a C-terminal carboxylate. The axial ligands of copper(II) are water, one of which forms a bridge between copper and the sidechain and amide of N3. This interaction represents a crystal contact and coordination differs from the GHK peptide in the axial waters and N3 participating in a water bridge to the metal ion (Fig. 2d)^46^. The difference between the two structures suggests the third position can be variable. Given the proximity of the N-terminus to the inner leaflet (Fig. 2b and Supplementary Fig. 11), the copper(II) bound structure could engage lipid headgroup, resulting in enhanced lipid binding affinity. Analysis of ABC transporter sequences, reveals more than 400 proteins contain a histidine in the second position, including some with an N-terminal sequence of MHK, and may have relevance for other ABC transporters.

### Characterizing lipid binding to vanadate-trapped MsbA

**Fig. 3:**
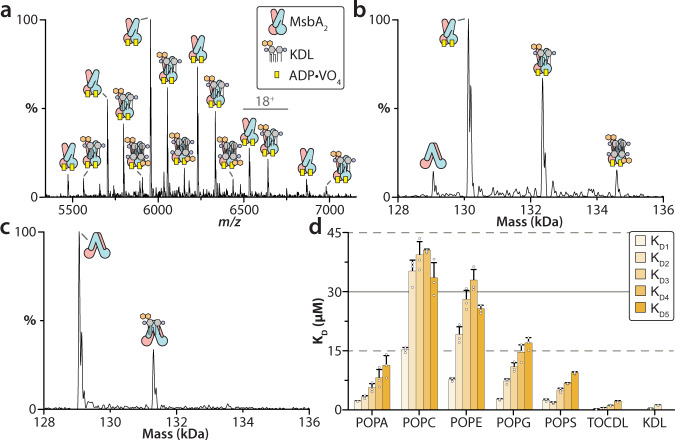
Biophysical characterization of lipid binding to MsbA trapped with ADP and vanadate. **a** Representative mass spectrum of MsbA trapped with ADP·VO_4_ and in the presence of 0.4 μM KDL. **b** Deconvolution of the mass spectrum shown in panel **a**. **c** Deconvoluted mass spectrum of non-trapped MsbA in the presence of the same amount of KDL. A significant reduction in KDL binding to MsbA is observed. **d** K_D_ values for individual lipid binding events to MsbA trapped with ADP and vanadate. Reported are the mean and standard deviation (*n*=3, biological replicates). Source data are provided as a Source Data file.

To determine the impact of MsbA conformation on lipid binding affinity, analogous experiments were performed using MsbA trapped in an open, OF conformation with adenosine diphosphate (ADP) and vanadate (VO_4_). The native mass measurement show that each subunit of the transporter is bound to copper(II), ADP, and VO_4_ molecules (Fig. 3a and Supplementary Table 1). The dissociation constants revealed that MsbA in the open, OF conformation displayed higher lipid binding affinity for a subset of lipids (Fig. 3d and Supplementary Fig. 12–14, and Supplementary Table 7). For example, in the presence of 0.4 μM of KDL, vanadate-trapped MsbA binds up to two lipids whereas the non-trapped protein binds only one KDL (Fig. 3b–c). In particular, the binding affinity for KDL (K_D1_ = 0.3 µM) significantly increased by two-fold compared to the non-trapped protein (Fig. 3d). Interestingly, the change in K_D_ for each subsequent lipid binding event was significantly reduced, an indication of strong positive cooperativity. The binding of POPC and POPE are reminiscent of that for MsbA partially loaded with copper(II). POPA and POPG displayed an overall modest increase in binding affinity. Together, these results demonstrate that different conformational states of MsbA bind lipids with different affinities.

### CryoEM structure of vanadate-trapped MsbA in complex with KDL

**Fig. 4:**
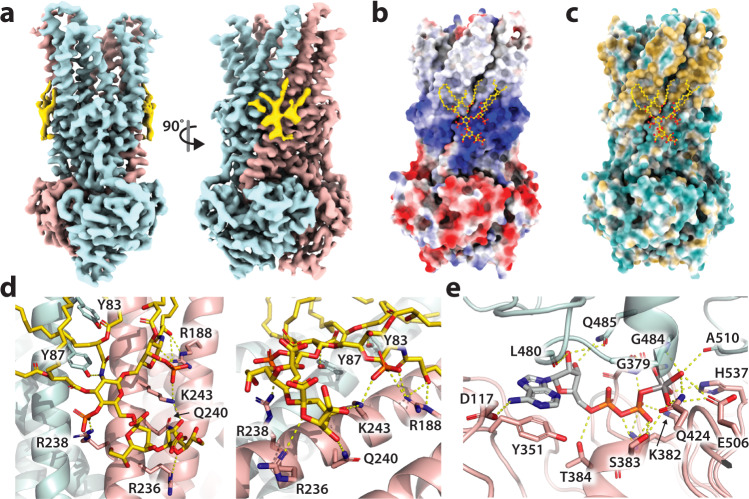
Structure of vanadate-trapped MsbA reveals a distinct, exterior KDL binding site. **a** Cryo-EM reconstruction (3.6 Å) of MsbA(ADP·VO_4_) in complex with KDL. The density for KDL is shown in yellow, and MsbA subunits are shown in pink and blue. **b** Coulombic electrostatic potential (scale bar −10 to +10 as computed by ChimeraX^62^) is colored red and blue for negative and positive charges, respectively. KDL and interacting residues are shown in ball and stick representation. **c** MsbA shown with hydrophilic and hydrophobic surfaces colored blue and gold, respectively. **d** Different views of KDL bound to MsbA. KDL and interacting residues shown in stick representation. Bonds are shown as dashed yellow lines. Residues are labelled. **e** View of the bound ADP·VO_4_ and interacting residues shown as described in **d**.

**Fig. 5:**
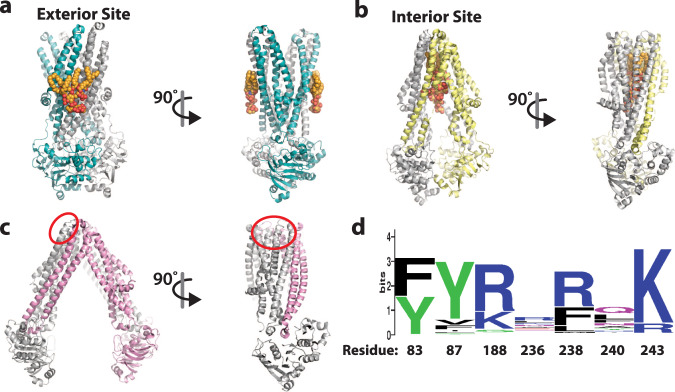
Comparison of MsbA LPS binding sites. Structures are shown in cartoon representation with lipid (if present) shown in orange spheres. Two views are shown (0 and 90°). Shown is the **a** exterior binding site (this work), **b** interior binding site (PDB 6BPL)^6,17^, and **c** putative exterior binding site (PDB 6BL6)^19,64^. In panel **c**, the density was not clear enough to model the lipid and putative site is denoted by a red circle^19^. **d** Sequence logo of KDL interacting residues (83, 87, 188, 236, 238, 240, and 243) based on an alignment of 258 MsbA sequences from across the bacterial phylogeny.

As KDL binds copper(II)-bound MsbA with an affinity greater than the other lipids, we prepared MsbA trapped with ADP and vanadate (62 µM) in the presence of two-fold molar excess of KDL (124 µM) in C_10_E_5_ for cryo-electron microscopy (cryoEM) studies. The structure of the complex was determined to a resolution of 3.6 Å (Fig. 4a, Supplementary Figs. 15–16, Supplementary Table 8, and Supplementary Note 2). The structure is similar to that of the vanadate-trapped MsbA from *S. typhimurium* but differs from the previously reported vanadate-trapped, occluded MsbA structures^17,47^, largely in the TMD (Supplementary Fig. 17). Additional density is observed centered on TM5 in a region within the cytoplasmic leaflet of the inner membrane, in which KDL could be directly modeled into this density (Fig. 4a). The location of KDL is different from the interior binding site^6,17^ and the putative, exterior binding site^19^ located on the opposite side of the inner membrane (Fig. 5). The electrostatic surface of MsbA shows that the headgroup of KDL is bound to a large, basic patch (Fig. 4b). Acyl chains of KDL could be modeled, which interact with the hydrophobic surface of MsbA (Fig. 4c). Extensive interactions are formed between KDL and MsbA with an interface area of 2779 Å^2^. The characteristic phosphoglucosamine (P-GlcN) substituents of LOS are coordinated by R238 on one side, and R188 and K243 on the other side (Fig. 4d). In addition, R236, Q240, and K243 interact with one of the Kdo groups of LOS (Fig. 4d). We also note that the concentration of KDL used here is much less than that used for the structures of MsbA bound G907 or TBT1, in which 1 mM and 400 µM of the respective drug was used^6,8^. This further strengthens the specific binding of KDL to the exterior site. In addition, there is clear density for ADP·VO_4_ in the NBDs, coordinated by a network of conserved residues (Fig. 4e and Supplementary Fig. 18). In short, the exterior KDL binding site identified here may have role in regulating MsbA function.

### Implications of the exterior KDL binding site in MsbA

The unique KDL binding site uncovered here reveals an evolutionarily conserved feature of MsbA. The residues (R188, R238, and K243) that engage the characteristic P-GlcN substituents of LOS are conserved (Fig. 5d). Similar interactions have been observed for the interior LOS site in MsbA^6,17^ and recognition of LPS in LptB2FG, an LPS ABC transporter^48^. Other residues (R236 and Q240) that coordinate a Kdo group of KDL are also conserved (Fig. 5d). As most LOS of gram-negative bacteria synthesize a KDL molecule resembling those found in *E. coli*^3^, the conservation of residues and their interaction with KDL establish this as a unique feature of MsbA compared to other transporters. Moreover, the large, basic patch that nestles the KDL headgroup extends beyond the Kdo groups that could further engage the core oligosaccharide of LOS, the glycolipid MsbA flips. This basic patch likely extends to other MsbAs and important for recognition of LOS, including other lipids, that vary in structure in different bacteria^2^.

### Probing the exterior KDL binding sites

**Fig. 6:**
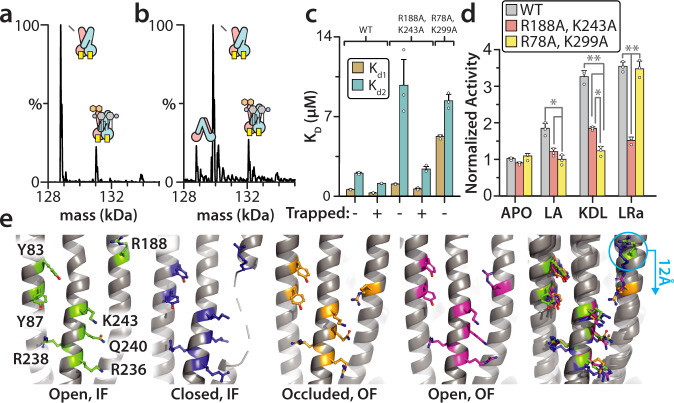
Characterization of the exterior KDL binding site. **a** Deconvoluted mass spectrum of 0.3 μM MsbA^R188A,K243A^ in the presence of 0.4 μM KDL. **b** Deconvoluted mass spectrum of 0.4 μM vanadate-trapped MsbA^R188A,K243A^ with same concentration of KDL as in panel **a**. **c** K_D_ values for KDL binding to the wild-type and mutant MsbA. **d** ATPase activity of MsbA, MsbA^R188A,K243A^, and MsbA^R78A,K299A^ in the presence and absence of 5 μM lipid A (LA) (^*^p = 0.047; 0.011), KDL (^**^p =  0.008; 0.005, ^*^p = 0.019), or Ra-LPS (LRa) (^**^p = 0.004; 0.009). A two-sided student’s *t*-test was used to test for statistical significance. **e** Alignment of different structures to a region of TM5, residue range from 230 to 250. The first four panels shown (from left to right) correspond to apo (this report), G907 bound (PDB 6BPL), and vanadate-trapped in an occluded (PDB 7BCW) or open (this report) MsbA structures. The rightmost panel is an overlay of all four structures. Reported are the mean and standard deviation (*n*=3, biological replicates). Source data are provided as a Source Data file.

A series of MsbA mutants engineered to impact KDL binding were evaluated. Some of the MsbA mutant proteins could be expressed and purified but were biochemically unstable where truncation of the transporter was observed after treatment with vanadate, such as MsbA^Y87F,R238A^ (Supplementary Figure 19). MsbA containing the R188A and K243A mutations (MsbA^R188A,K243A^), engineered to disrupt the interaction with one of the P-GlcN substituents of KDL, was biochemically stable and suitable for determining K_D_s (Fig. 6a–c and Supplementary Fig. 20). For the non-trapped protein, K_D1_ increased two-fold and K_D2_ increased five-fold. In the trapped state, K_D1_ and K_D2_ also both statistically increased by more than two-fold. As MsbA is known to be stimulated by some lipids^22–24,49^, we determined the ATPase activity of MsbA in the absence and presence of lipid (Fig. 6d). Wild-type MsbA was stimulated by KDL at levels observed by others^17,22^. However, RaLPS (LRa), an LOS with a complete *E. coli* R2-type core^50^, stimulated MsbA ATPase activity to a higher degree (Fig. 6d)^22^. Consistent with a previous report^22^, lipid A (LA), similar to KDL but lacking the Kdo substituents, stimulated MsbA but to lesser extent, highlighting the importance of the Kdo groups. MsbA^R188A,K243A^ displayed a statistically significant decrease in stimulation that was independent of the lipid type (Fig. 6d). Lipid-induced stimulation of MsbA containing R78A and K299A mutations (MsbA^R78A,K299A^), engineered to disrupt binding at the interior site (Fig. 5b), was also assessed. MsbA^R78A,K299A^ showed no stimulation by LA and KDL but activity was stimulated by LRa to the same level as the wild-type protein (Fig. 6d). Next, we inspected the residues coordinating KDL in MsbA structures (Fig. 6e and Supplementary Fig. 21). Except for R188, the KDL interacting residues are in similar positions. However, in (occluded and open) OF states, TM4 is displaced ~12 Å in a direction toward the other residues, priming R188 to engage the P-GlcN of KDL. This additional interaction explains the enhancement in KDL binding affinity. Together, these results indicate that the exterior KDL binding site has a direct role in allosterically stimulating MsbA ATPase activity.

In summary, native mass spectrometry data reveal that MsbA co-purifies with copper(II), an observation that would remain unnoticed using traditional methods, and protein-lipid interactions are directly influenced by copper(II) binding as well as the conformation of the transporter. Structural studies show the N-terminus of MsbA coordinates copper(II) in a similar fashion as the GHK peptide. Copper(II) binding to MsbA impacts lipid binding, and the N-terminus is located near the bilayer where it plausibly interacts with lipid headgroups. More broadly, copper(II) binding may have a regulatory role, such as coupling copper(II) levels with LPS biogenesis, and warrants further investigation. Another structure illuminates a distinct, exterior KDL binding site is an allosteric modulator of ATPase activity and a conserved feature of MsbA. Mutagenesis studies document that this exterior, allosteric site is also sensitive to hexaacylated lipid A species, such as LA and LRa. These results provide compelling evidence of a feedforward activation mechanism for MsbA, a rare principal of control in biosynthetic pathways that is best described for pyruvate kinase^51^, tuning MsbA activity to match cellular production of cytoplasmic LOS and precursors thereof.

## Methods

### Construction of MsbA and other expression plasmids

The *msbA* gene (UniProt P60752) and pCDF-1b plasmid (Novagen) were amplified by polymerase chain reaction (PCR) using Q5 High-Fidelity DNA Polymerase (New England Biolabs, NEB) from *Escherichia coli* genomic DNA and purified plasmid, respectively. Primers were designed using the online NEBuilder Assembly Tool (NEB) and amplified products were gel purified prior to HiFi DNA Assembly (NEB) following the manufacturer’s protocol. The resulting construct, pCDF-MsbA, expressed MsbA with an N-terminal TEV cleavable His_6_ fusion protein. To generate mutant forms of MsbA, primers were designed using online tool NEBaseChanger (NEB) and mutants introduced using the KLD enzyme mix (NEB) following the manufacturers protocol. MsbA was also cloned into a modified pET15 plasmid to express MsbA with a HRV3C protease cleavable C-terminal fusion to superfolder GFP^52^ followed by a 6x His tag. The N-terminal sequence of MsbA was grafted onto MBP, superfolder GFP^52^ and T4 lysozyme by subcloning into the pCDF-MsbA plasmid and keeping residues 1-8 of MsbA. A similar fusion strategy has been done for the GHK peptide^53^. Truncation of the MsbA N-terminus was carried using KLD enzyme mix (NEB) following manufacturers protocol. The GFP fusion for successful structure determination had a N-terminal sequence after TEV protease cleavage of MHNDKGEELF with the MsbA sequence underlined. All plasmids were confirmed by DNA sequencing. Primers used in this study can be found in the Source Data File.

### MsbA expression and membrane preparation

The wild-type and mutant MsbA expression plasmids were transformed into *E. coli* (DE3) BL21-AI competent cells (Invitrogen) and incubated at 37 °C until the OD_600nm_ ≈ 0.6-1.0 at which point the cultures were induced with final concentrations of 0.5 mM IPTG (isopropyl β-D-1-thiogalactopryanoside) and 0.2% (w/v) arabinose. The cultures were induced overnight at 25 °C. The cultures were then harvested at 4500 × g for 12 min and the resulting pellet was resuspended in 20 mM Tris, 300 mM NaCl, pH 7.4 and supplemented with Roche cOmplete Protease Inhibitor Cocktail tablet. The suspension was lysed in a Microfluidics M-110P microfluidizer operating at 25,000 psi on ice. The lysate was centrifuged at 40,000 × *g* for 20 min and the resulting supernatant was centrifuged at 100,000 × *g* for 2 h. The resulting pellets were collected and homogenized in 20 mM Tris, 150 mM NaCl, 20% (v/v) glycerol, pH 7.4. The membrane solution was extracted with 1% (w/v) DDM, rotating overnight at 4 °C. The extraction was then centrifuged at 40,000 × *g* for 10 min and the resulting supernatant was supplemented with 10 mM imidazole and filtered with a 0.45 µm syringe filter.

### Detergent screening and optimization of purification

The extracted material was subjected to extensive detergent screening^37^ to determine the delipidating ability of each detergent on MsbA. In short, His-tagged MsbA was bound to 100 µL Ni-NTA beads (Qiagen) equilibrated with NHA buffer (20 mM Tris, 150 mM NaCl, 10 mM imidazole, 10% (v/v) glycerol, pH 7.4) supplemented with 2x the critical micelle concentration (CMC) of DDM and then washed with 5 column volumes (CV) of NHA containing 2x CMC DDM buffer. The bound protein was then treated with 10 CV of NHA containing 2x CMC DDM buffers supplemented with 10x CMC of various detergents (Anatrace). The column was then re-equilibrated with 5 CV of NHA-2x CMC DDM buffer and eluted with 2 CV of NHA containing 2x CMC DDM buffer supplemented with 500 mM imidazole. To check the degree of delipidation via native mass spectrometry, the eluent was buffer exchanged into 200 mM ammonium acetate, 2x CMC DDM, pH 7.4 via Micro Bio-Spin P-6 gel centrifuge columns (Biorad) following the manufacturer’s protocol. After determination and purification with the optimal detergent wash (NG), the protein was buffer exchanged back into NHA-2x CMC DDM on a HiPrep 26/10 desalting column (GE Healthcare). The sample was then treated with TEV protease, produced in-house, overnight at room temperature to remove the N-terminal His tag, 10 mM β-mercaptoethanol was added during the TEV treatment. The digested material was passed over Ni-NTA agarose equilibrated with NHA-2x CMC DDM and the flow-through containing the cleaved material was collected. The material was concentrated using a centrifugal concentrator (Millipore, 100 kDa molecular weight cutoff) followed by injection onto a Superdex 200 Increase 10/300 GL (GE Healthcare) column equilibrated with 20 mM HEPES, 200 mM NaCl, 10% (v/v) glycerol and 2x CMC C_10_E_5_. Peak fractions containing dimeric MsbA were pooled and flash frozen at −80 °C.

### Preparation of MsbA for native MS and structural studies

For MsbA with reduced copper(II) binding, additional 1 mM MgCl_2_ was added to all buffer and MsbA solution in the purification step using Ni-NTA beads. Copper(II) saturated MsbA was obtained by adding 20 uM copper (II) acetate then buffer exchanged using Bio-Spin column to remove excess copper(II). MsbA trapped by vanadate was obtained by adding ATP and MgCl_2_ to MsbA to reach the final concentration of 10 mM for both then incubating at room temperature for 10 min. After incubation, vanadate (pH 10) was added to reach final concentration of 1 uM followed by incubation at 37 °C for 10 min. MsbA samples was buffer exchanged using Bio-Spin column to 200 mM ammonium acetate supplemented with 2x CMC C_10_E_5_ for native MS studies. To prepare MsbA samples for Cryo-EM studies, MsbA and trapped MsbA were preloaded with copper(II) then purified by Superdex 200 Increase 10/300 GL size exclusion column equilibrated with 200 mM NaCl, 20 mM HEPES and 2x CMC C_10_E_5_ without glycerol. Peak fractions containing MsbA were pooled and concentrated to 8 mg/mL then mixed with KDL at 1:2 molar ratio (1 KDL for 1 MsbA subunit).

### Native mass spectrometry

Samples were loaded into gold-coated glass capillaries made in-house^37^ and were ionized via electrospray into a Thermo Scientific Exactive Plus Orbitrap with Extended Mass Range (EMR). For native mass analysis, the instrument was tuned as follow: source DC offset of 25, injection flatapole DC to 8.0 V, inter flatapole lens to 7, bent flatapole DC to 6.0, transfer multipole DC to 2 and C trap entrance lens to 2, trapping gas pressure to 6.0 with the in-source CID to 60.0 eV and CE to 100, spray voltage to 1.70 kV, capillary temperature to 200 °C, maximum inject time to 200 ms. Mass spectra were acquired with setting of 17,500 resolution, microscans of 1 and averaging of 100.

### Determination MsbA-lipid equilibrium binding constants

Lipids were prepared as previously described^54^, in which lipids dissolved in chloroform were dried under nitrogen flow placed under vacuum overnight followed by dissolving in water. The concentration of MsbA was determined using a DC protein assay (BioRad) with bovine serum albumin as the standard. MsbA was incubated with lipids at varying concentrations and mixed with 200 mM ammonium acetate supplemented with 2x CMC lauryldimethylamine oxide (LDAO) at 1:1 volume ratio. Samples were incubated in the nano electrospray ionization source chamber for a minute to reach equilibrium prior to data acquisition. These samples analyzed on Orbitrap Exactive Plus EMR mass spectrometer (Thermo Scientific) operating at identical settings as described above. The mass spectra for each titration event were obtained in triplicate. The mass spectra were deconvoluted using UniDec^55^ and the resulting peak intensities for apo and lipid-bound protein were determined. The relative abundance for each species was determined by dividing the peak intensity by the total intensity to convert to mole fraction for each independent experiment. For MsbA (P) binding the *N*^*th*^ lipid (L_n_), we applied the following sequential lipid binding model:

$${P{L}_{n-1}}+L{\mathop{\Longleftrightarrow }\limits^{{K}_{A}}}P{{L}_{n}}$$

where:

$${K}_{{An}}=\frac{\left[P{L}_{n}\right]}{[P{L}_{n-1}]\left[L\right]}$$

To calculate the mole fraction of a particular species^54^:

$${F}_{{PLn}}=\frac{{\left[L\right]}_{{free}}^{n}{\prod }_{j=1}^{n}{K}_{{Aj}}}{1+{\sum }_{i=1}^{n}{\left[L\right]}_{{free}}^{i}{\prod }_{j=1}^{n}{K}_{{Aj}}}$$

For each titrant in the titration the free concentration of lipid was computed as follows:

$${\left[L\right]}_{{free}}={\left[L\right]}_{{total}}-{\left[P\right]}_{{total}}\mathop{\sum }\limits_{i=0}^{n}i{F}_{{PLi}}$$

The sequential lipid binding model was globally fit to the mole fraction data by minimization of pseudo-${\chi }^{2}$ function:

$${\chi }^{2}=\mathop{\sum}\limits_{j=1}^{m}\mathop{\sum}\limits_{k=1}^{d}{({F}_{i,\,j,{\exp }}-{F}_{i,\,j,{calc}})}^{2}$$

where *n* is the number of bound ligands and *d* is the number of the experimental mole fraction data points.

### Determination of metal bound to MsbA

Samples of MsbA were submitted to the Elemental Analysis Laboratory at Texas A&M University for elemental analysis by inductively coupled plasma mass spectrometry. A NexION ICP mass spectrometer (PerkinElmer) was used with operating parameters provided in Supplementary Table 2.

### MsbA liposome preparation

POPC (Avanti) was resuspended in chloroform and dried under a stream of nitrogen gas. The film was washed with pentane and dried under a stream of nitrogen gas again. The lipid film was stored in a desiccator overnight and rehydrated to a final concentration of 20 mM in rehydration buffer (20 mM HEPES pH 7.4, 150 mM KCl), occasionally agitated for an hour and then stored at −80 °C. The liposome mixture (~150 μL) was diluted by half with the rehydration buffer and extruded using a mini-extruder (Avanti Polar Lipids) with a 100 nm polycarbonate membrane until the solution became translucent. The extruded liposomes were then separated into two different portions of equal volume: one for the wildtype protein and one for the mutant. The extruded liposomes were then solubilized with an equal volume of solubilization buffer (20 mM HEPES, pH 7.4, 150 mM KCl and 20 mM DDM) and rotated at room temperature for 30 min or until clear. The MsbA samples were then added at a protein to lipid ratio of 1:100 (w/w) and rotated for an hour at room temperature. BioBeads were added to the liposomes and rotated overnight at 4 °C to remove detergent.

### MsbA activity assay

The ATPase activity of MsbA was determined following a modified version of the malachite green assay^56^. For the fully delipidated (apo) protein, 400 nM of MsbA (in 200 mM ammonium acetate supplemented with 1x CMC C_10_E_5_ and 1x CMC LDAO) was incubated with 5 mM MgCl_2_ and 200 μM ATP at 37 °C for 12 min. For analysis of protein with KDL, lipid was added to the sample at a final concentration of 5 µM. Endpoint samples were collected at 3, 6, 9, 12 min and stopped with the addition of malachite green solution with the following components: 3:1 mixture of 0.045% (w/v) malachite green and 4.2% (w/v) ammonium molybdate prepared in 4 N HCl, 0.04% (v/v) Triton X-100 (final concentration). Then 34% (w/v) sodium citrate was added to stop the coloring reaction. The quenched reactions were incubated at room temperature for 30 min and absorbance at 650 nm was measured on a CLARIOstar plate reader (BMG LabTech). The hydrolyzing rate of ATP was obtained by plotting the slope of absorbance of samples collected at 3, 6, 9, and 12 min.

### X-ray structure of N-terminal peptide bound to copper(II)

Initial crystallization trials were carried out for the GFP fusion protein at a concentration of 1 mM (using an extinction coefficient at 490 nm of 39.2 × 10^3^ M^−1^ cm^−1^)^52^ using a Mosquito LCP (TTP Labtech) crystallization robot in hanging drop plates at 20 °C. Crystals grew in index condition C5 (60% Tacsimate pH 7.0) and were further optimized by increasing the concentration of Tacsimate pH 7.0 to 70%. Crystals were cryoprotected using 100% Tacsimate pH 7.0. Single crystals were mounted with CrystalCap HT Cryoloops (Hampton Research) prior to flash freezing in liquid nitrogen. The initial diffraction data were collected in-house on a Rigaku Raxis-IV++. Initial phases were determined using molecular replacement with PDB code 2B3P. Model refinement and building were performed using Phenix^57^ and Coot^58^. Anomalous data was collected using a wavelength of 1.378 Å at the Advanced Photon Source on beamline 24-ID-C. Although the structure could be determined by SAD phasing using Phenix AutoSol program, the model built from the in-house data was used for molecular replacement followed by model building and refinement.

### Sample preparation for single-particle cryo-EM

Vitrification was performed using a Vitrobot Mark IV (Thermo Fisher) operating at 8 °C and 100% humidity. A total of 3.5 μL of sample (8 mg/mL copper-loaded MsbA either apo or trapped with ADP-vanadate in 200 mM NaCl, 20 mM HEPES pH 7.4, supplemented with 2x CMC C_10_E_5_) was applied to holey carbon grids (Quantifoil 300 mesh Cu 1.2/1.3) glow-discharged for 30 s. Samples contained two-fold molar excess of KDL. The grids were blotted for 5 s at blotting force 1 using standard Vitrobot filter paper (Ted Pella, 47000-100), and then plunged into liquid ethane.

### Data collection for single-particle cryo-EM

The optimized grids were sent to the Advanced Electron Microscopy Facility at the University of Chicago for data collection. The dataset was collected as movie stacks with a Titan Krios electron microscope operating at 300 kV, equipped with a K3 direct detector camera. Images were recorded at a nominal magnification of 81,000x at super-resolution counting mode by image shift. The total exposure time was set to 4 s with a frame recorded every 0.1 s, resulting in 40 frames in a single stack with a total exposure around 50 electrons/Å^2^. The defocus range was set at −1.0 to −2.5 μm. See Supplementary Table 8 for the details of data collection parameters.

### Image processing for single-particle cryo-EM

Collected movies were subjected to motion correction by MotionCor2^59^. Subsequent processing was carried out in cryoSPARC^60^. The detailed data processing flow is shown in Supplementary Fig. 15 (vanadate-trapped MsbA) and Supplementary Fig. 22 (open, inward-facing MsbA). Stage drift and anisotropic motion of the stack images were first corrected by patch-based motion correction. CTF parameters for each micrograph were determined by patch-based CTF estimation. For vanadate-trapped MsbA, the particles were picked using the templates generated from the blob picker. For the open, inward-facing MsbA, the final particle set was picked using templates generated from a 3D model from an earlier reconstruction. For both datasets, the particles were cleaned by two rounds of 2D classification. Three initial models were generated from the remaining particles using ab initio reconstruction. The particles were further classified by heterogeneous refinement based on three initial models. For the vanadate-trapped MsbA, the best class of particles was selected for a non-uniform refinement with per-particle defocus and CTF optimization, and a C2 symmetry imposed, resulting in a final map resolved at 3.6 Å. For the open MsbA, the best class of particles was selected for a non-uniform refinement with either C2 or C1 symmetry imposed, resulting in final maps resolved at 3.88 Å and 4.06 Å, respectively. See Supplementary Table 8 for the details of image processing statistics.

### Model building, refinement, and validation for single-particle cryo-EM structures

The previously reported structure^17^ of MsbA with ADP-vanadate from *Escherichia coli* (PDB 5TTP) was docked into the cryo-EM map using Chimera^61^. The model was manually refined using Coot^58^. Phenix^57^ was used to generate the coordinates and restraint files for KDL. The final model underwent multiple rounds of real-space refinement using Phenix with secondary-structure and Ramachandran restraints. Geometry outliers were manually fixed in Coot (after each round). The statistics of the final round of model refinement and the model geometry are reported in Supplementary Table 9. Figures were generated using ChimeraX^62^ and Pymol (Schrödinger LLC., version 2.1). See Supplementary Table 9 for the details of model statistics.

### Conservation and sequence analysis of bacterial MsbA

Sequences for conservation analysis were gathered using NCBI Blast and the *E. coli* MSBA protein as input. We separately searched for MSBA sequences from gammaproteobacteria, deltaproteobacteria, alphaproteobacteria, epsilonproteobacteria, the FCB clade, and Bacilli to get broad representation across Bacteria. We then aligned the sequences with Muscle 3.83. We removed all gaps caused by sequence absent in the *E. coli* sequence and extracted sites interfacing with KDL using a custom python script. A subalignment containing only these sites was used with the weblogo server (https://weblogo.berkeley.edu/) to generate the sequence logo. For analysis of N-terminal sequences of ABC transporters, more than >20 k MsbA sequences were downloaded from UniProt. A python script making use of BioPython^63^ analyzed sequences containing an N-terminal sequence of to begin with MH.

### Reporting summary

Further information on research design is available in the Nature Portfolio Reporting Summary linked to this article.

## Supplementary information

Supplementary Information

Reporting Summary

## Data Availability

Atomic coordinates and structure factors for the crystal structure of an N-terminal fragment of MsbA fused to GFP and bound to copper(II) has been deposited in the Protein Data Bank (PDB) under accession code 8DHY (copper(II)-bound MsbA N-terminal peptide fused to GFP). MsbA cryoEM structures and maps have been deposited in the PDB and EMDB as follows: 8DMO (open, inward-facing MsbA) and EMD-27545 (open, inward-facing MsbA); and 8DMM (vanadate-trapped MsbA and bound to KDL) and EMD-27544 (vanadate-trapped MsbA and bound to KDL). Previously reported protein structures used in this study are: 5TTP (occluded, outward-facing MsbA); 6BPP (MsbA in complex with G092); 6BPL (MsbA in complex with G907 and LPS); 7BCW (vanadate-trapped MsbA); 6BL6 (open, inward-facing *Salmonella typhimurium* MsbA); 3B60 (open, outward-facing *Salmonella typhimurium* MsbA); and 2B3P (superfolder GFP). Native MS data has been deposited at Zenodo (10.5281/zenodo.7268757). A Source Data File is provided with this paper. Other data associated with this manuscript is available upon request.

## Source data

Source Data

## Code availability

Python code to determine individual equilibrium binding constants is available at https://github.com/LaganowskyLab.
