# Supplementary material for: Structural basis for lipid and copper regulation of the ABC transporter MsbA
Source: Nat Commun. 2022 Nov 26;13:7291. doi: 10.1038/s41467-022-34905-2 (PMC9701195; doi:10.1038/s41467-022-34905-2)
Supplement: Supplementary file 1 — Supplementary Information [file 41467_2022_34905_MOESM1_ESM.pdf]

Supplementary Information

Structural basis for lipid and copper regulation of the ABC transporter MsbA

Jixing Lyu,[1] Chang Liu,[2] Tianqi Zhang,[1] Samantha Schrecke,[1] Nicklaus P. Elam,[1] Charles Packianathan,[1,†] Georg Hochberg,[3] David Russell,[1] Minglei Zhao,[2] and Arthur Laganowsky[1,*]

[1] Department of Chemistry, Texas A&M University, College Station, TX 77843
[2] Department of Biochemistry and Molecular biology, University of Chicago, Chicago, IL 60637
[3] Max Planck Institute for Terrestrial Microbiology and Department of Chemistry, University of Marburg, Marburg, Germany
[†] Present address: Walter Reed Army Institute of Research, Pilot Bioproduction Facility, Silver Spring, MD 20910
*Corresponding author: ALaganowsky@chem.tamu.edu

Supplementary Note 1

Optimization of MsbA samples. An established detergent screening method was employed to optimize the purification of MsbA.[1] In brief, MsbA bound to nickel affinity resin was subsequently washed with various detergents. Of the detergents screened, those with short acyl chains (OG: n-octyl-β-D-glucopyranoside; OGNG: octyl glucose neopentyl glycol; LMNG: lauryl maltose neopentyl glycol) had the greatest impact on MsbA homogeneity. For example, the mass spectrum for MsbA washed with OG revealed mass spectral peaks corresponding to dimeric MsbA. In addition, the mass spectrum also indicates the presence of several adducts including a second charge state distribution that is ~3.5 kDa heavier in molecular weight. The adduct mass agrees with the mass of LPS, a lipid we found co-purifying with the mechanosensitive channel of large conductance.[2] LPS has been reported to co-purify with the transporter.[3,4] Pure samples of MsbA, devoid of contaminating lipids, was readily achieved by washing with n-nonyl-β-D-glucopyranoside (NG) followed by purification via size exclusion chromatography in the charge-reducing detergent pentaethylene glycol monodecyl ether ($C_{10}E_5$).[1,5] The use of charge-reducing detergents aids preservation of non-covalent interactions and native-like structure in the mass spectrometer.[5,6] These results indicate commonly prepared MsbA samples are heterogeneous, which poses significant challenges for employing high-resolution native MS to interrogate membrane protein-lipid interactions.

Supplementary Note 2

CryoEM structure of open, inward-facing MsbA. MsbA in the presence of two equivalents of KDL was subjected to cryo-electron microscopy (cryoEM) studies. The structure of MsbA was determined to a resolution of 3.9 Å (**Supplementary Figure 22-24 and Supplementary Table 8**). The maps were of sufficient quality to build the TMDs. However, density in some regions of the NBDs was less clear. MsbA adopts an open, inward facing conformation with the NDBs separated by ~57 Å from the Cα of R569 of one subunit to the other. In addition, tube-like density is observed in the central cavity and on outside, membrane facing side near residue 87 (**Supplementary Figure 23b**)**.** However, density was not clear enough to place the lipid.

Supplementary Figures

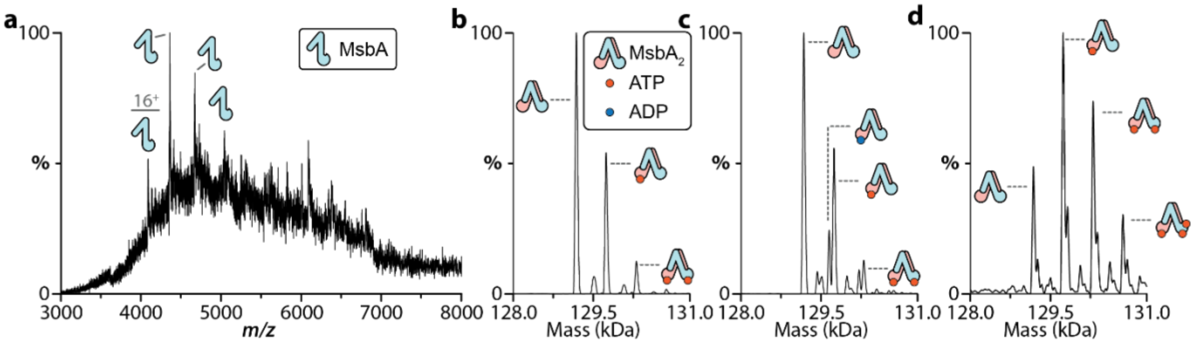

Supplementary Figure 1. Mass spectrum of MsbA purified in different detergents and ATP hydrolysis. a) Mass spectrum of MsbA purified in DDM following methods used for structural studies. The underlying broad hump indicates the sample is highly heterogeneous. Peaks corresponding to monomeric MsbA are result of dissociation of complex under the high energy instrument settings. b-d) Deconvoluted mass spectra of optimized MsbA samples in the presence of in the presence of 30 µM ATP collected b) after adding ATP and c) after 5h incubation on ice. ATP hydrolysis is evident by the binding of ADP and ATP to the transporter. d) The catalytically inactive MsbA^{E506Q} in the presence of 50 µM ATP did not hydrolyze ATP after incubation on ice for 2h.

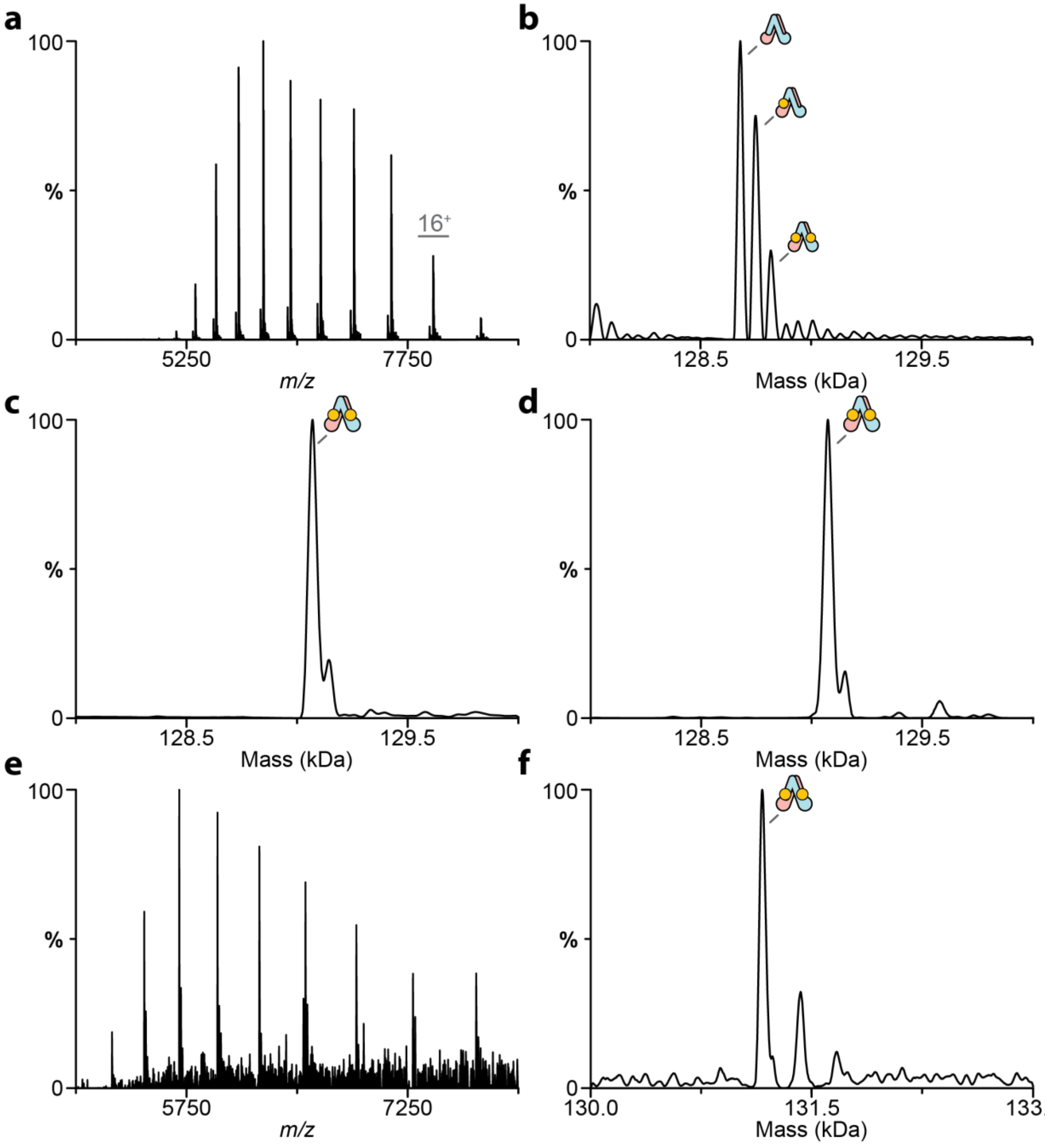

Supplementary Figure 2. Copper(II) binding to mutant MsbA(H562A, H576A) and ineffective chelation of copper(II) with trientine. a) Native mass spectrum of $MsbA^{H562A,H576A}$ as isolated. b) Deconvolution of the mass spectrum shown in panel a. Up to two copper(II) ions are bound to the mutant transporter. c-d) Deconvolution of wild-type MsbA after c) loading with copper(II) and d) after incubation with 2mM trientine. The specific chelator is unable to remove the MsbA bound copper(II). e) Native mass spectrum of MsbA expressed with C-terminal affinity tag and mixed with 5uM copper acetate. f) Deconvolution of the mass spectrum shown in panel e. The N-terminus of MsbA is intact and binds copper(II).

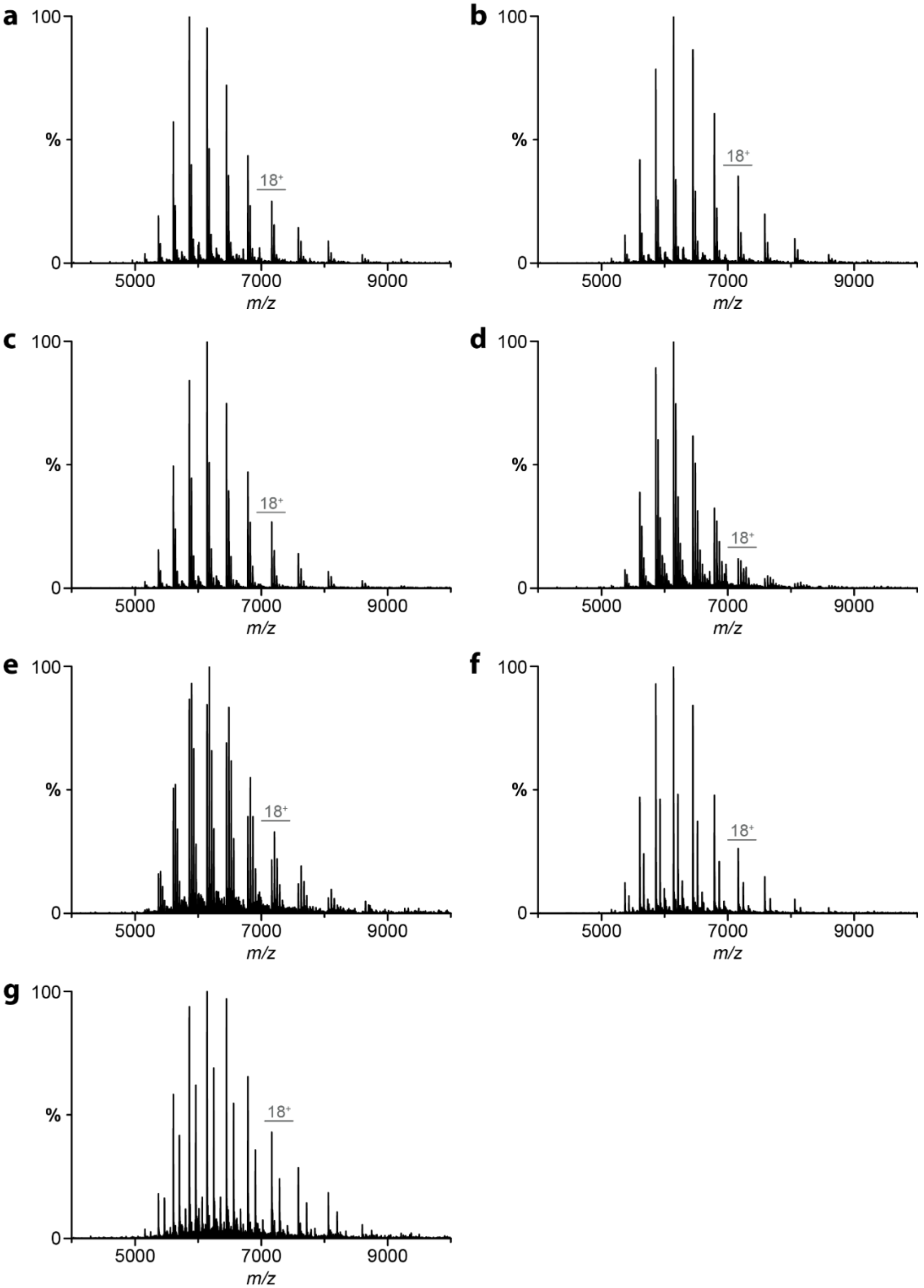

Supplementary Figure 3. Representative native mass spectra for lipid binding to MsbA partially loaded with copper(II). MsbA (0.5 µM) was mixed with 6 µM of a) POPA, b) POPC, c) POPE, d) POPG, or e) POPS and 1µM of f) TOCDL, g) KDL.

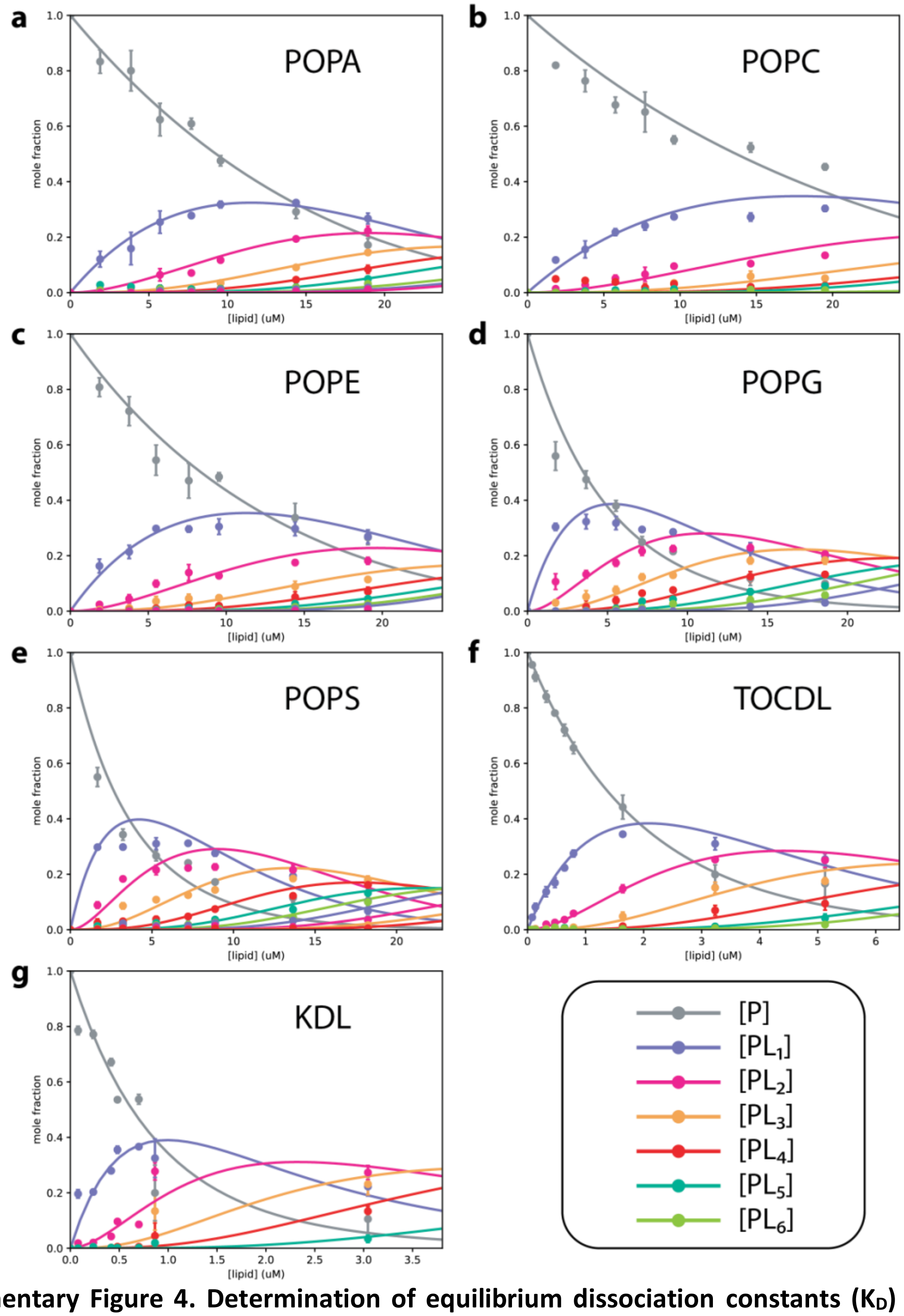

Supplementary Figure 4. Determination of equilibrium dissociation constants (K_D) for lipids binding MsbA partially loaded with copper(II). Plots of mole fraction for apo MsbA bound to different number of a) POPA, b) POPC, c) POPE, d) POPG, e) POPS, f) TOCDL, g) KDL and resulting fit from a sequential lipid-binding model (solid lines). The different lipids are labelled. [P] is the mole fraction of protein and [PL_x] is the mole fraction of protein with x lipids bound. Reported are the mean and standard deviation (*n* = 3 biologically independent samples). Source data are provided as a Source Data file.

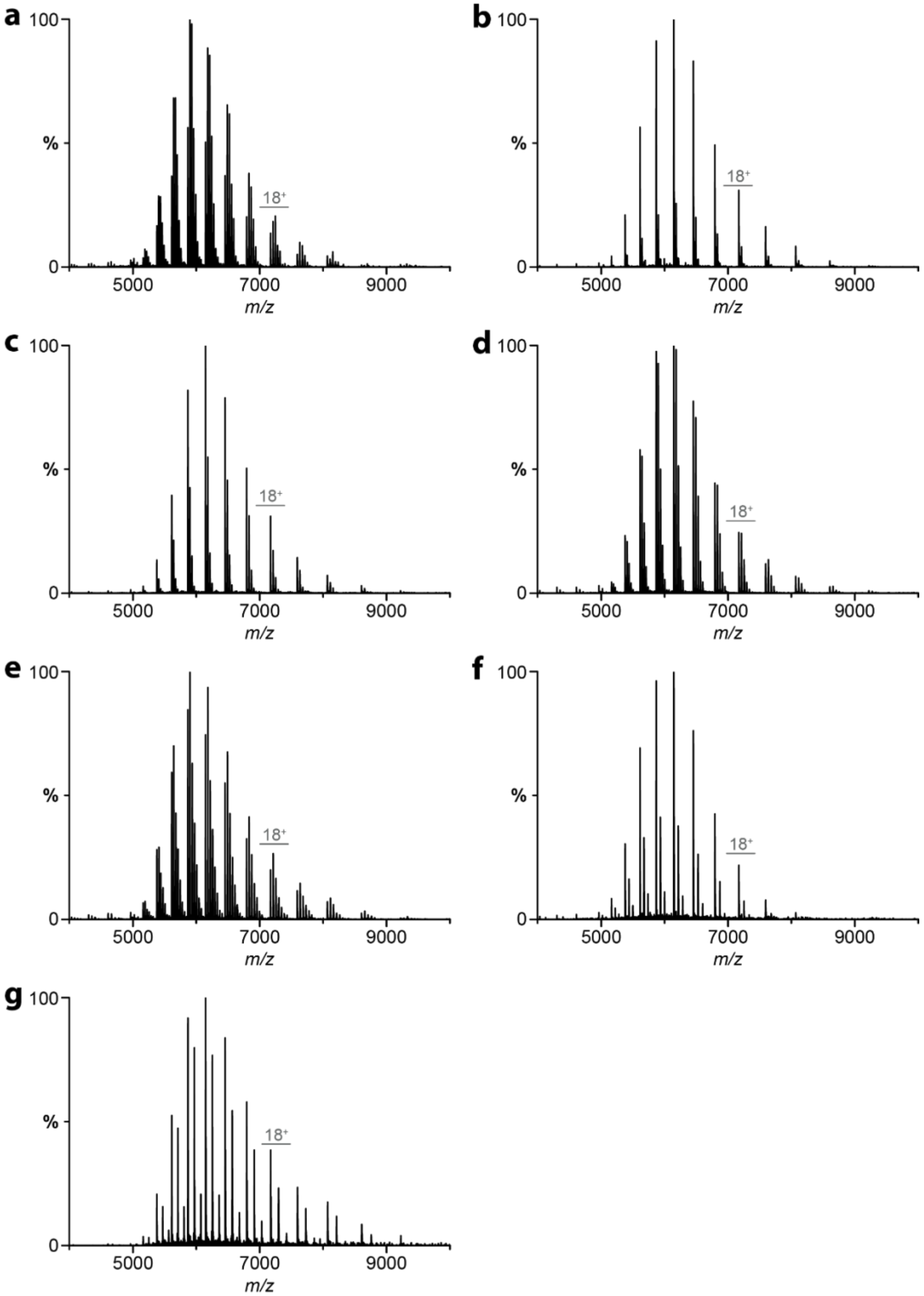

Supplementary Figure 5. Representative native mass spectra for lipid binding to MsbA fully loaded with copper(II). MsbA (0.5 µM) was mixed with 6 µM of a) POPA, b) POPC, c) POPE, d) POPG, e) POPS and 1µM of f) TOCDL. g) 0.43 µM. For KDL, the concentration is 0.7 µM of KDL.

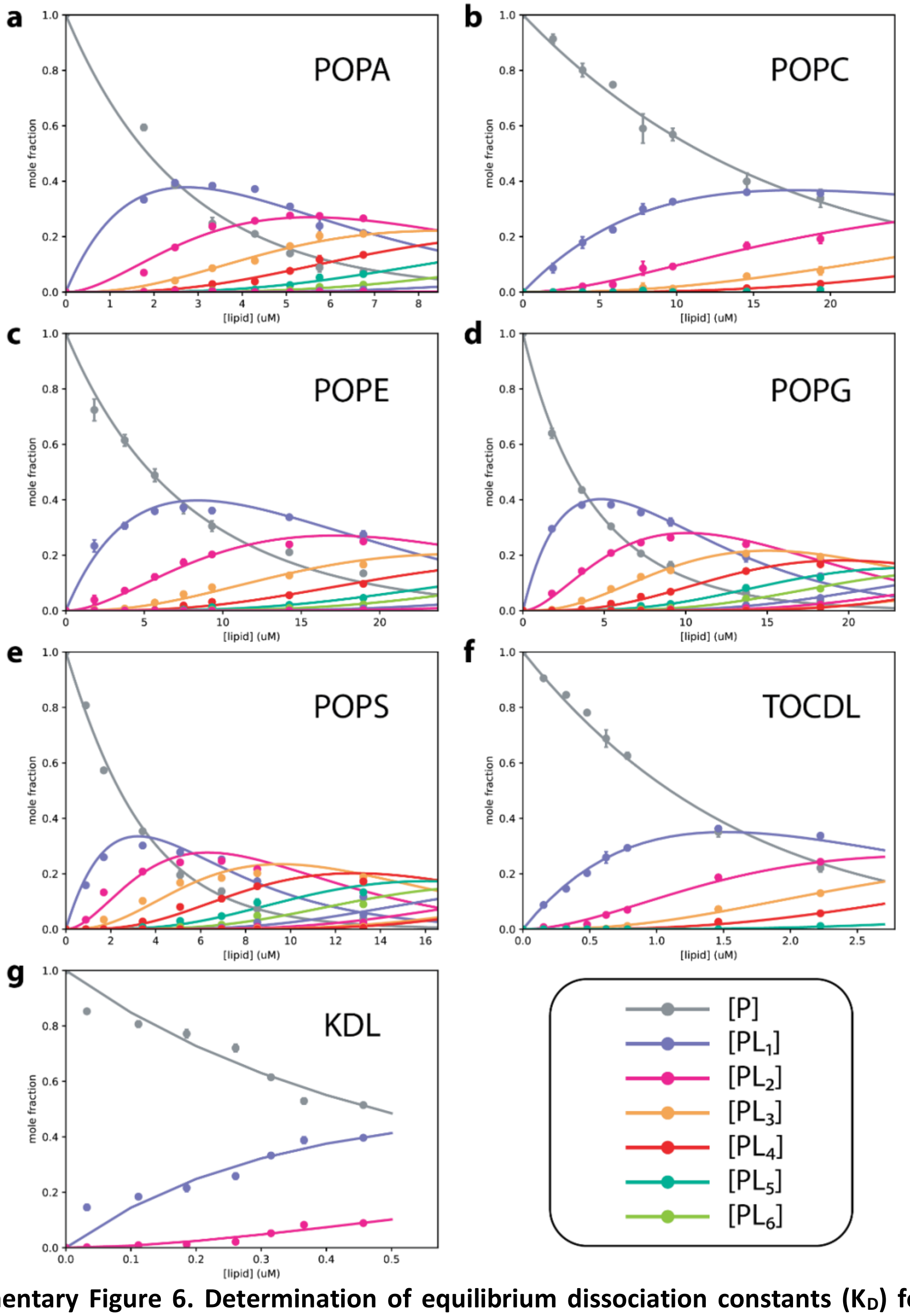

Supplementary Figure 6. Determination of equilibrium dissociation constants (K_D) for lipids binding MsbA fully loaded with copper(II). Plots of mole fraction for apo MsbA bound to different number of a) POPA, b) POPC, c) POPE, d) POPG, e) POPS, f) TOCDL, g) KDL and resulting fit from a sequential lipid-binding model (solid lines). The different lipids are labelled. [P] is the mole fraction of protein and $[PL_x]$ is the mole fraction of protein with x lipids bound. Reported are the mean and standard deviation (*n* = 3 biologically independent samples). Source data are provided as a Source Data file.

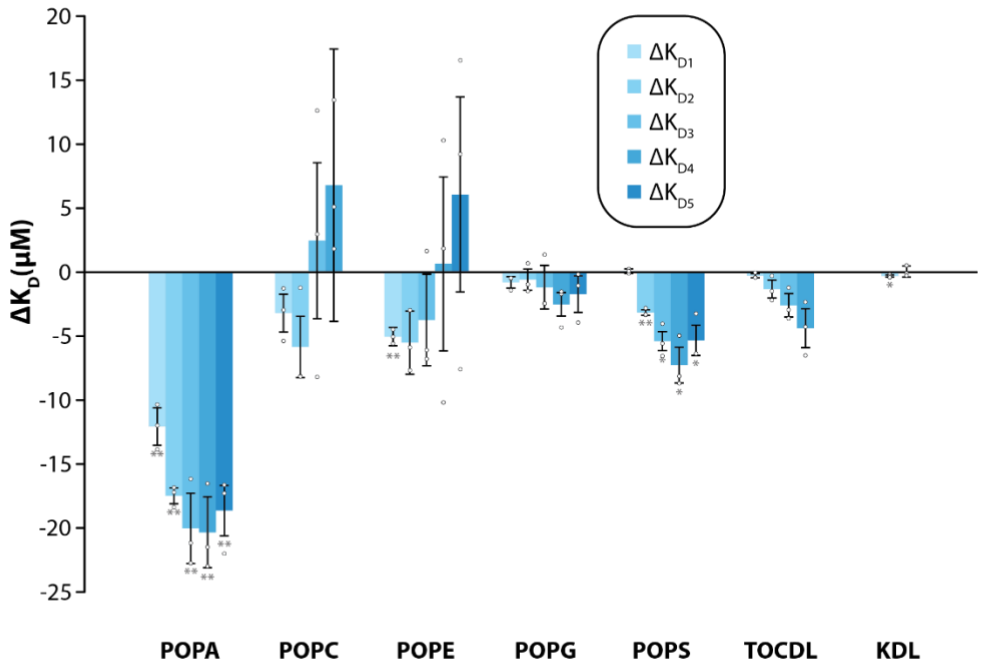

Supplementary Figure 7. Copper binding impacts MsbA-lipid interactions. Reported are the difference K_D values for MsbA saturated with copper(II) minus partially loaded. The asteriks denote student's t-test for MsbA partially versus fully loaded with copper(II). Reported are the mean and standard deviation (*n* = 3, biological replicates). Two-sided student's *t*-test (**p* < 0.05, ***p* < 0.01). *p*(POPA) = 0.007; 0.001; 0.010; 0.009; 0.008. *p*(POPE) = 0.004. *p*(POPS) = 0.003; 0.018; 0.025; 0.037. *p*(KDL) = 0.048. Source data are provided as a Source Data file.

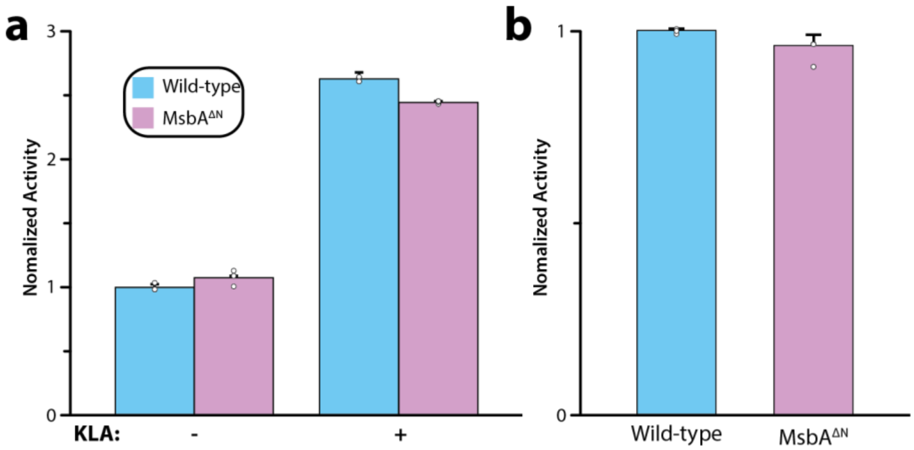

Supplementary Figure 8. ATPase activity for wild-type and N-terminal truncated MsbA. a) ATPase activity for MsbA samples in detergent $C_{10}E_5$, KDL was added to a final concentration of 5 µM. b) ATPase activity for MsbA samples assembled to POPC liposome. Reported are the mean and standard deviation (*n* = 3, biological replicates). Source data are provided as a Source Data file.

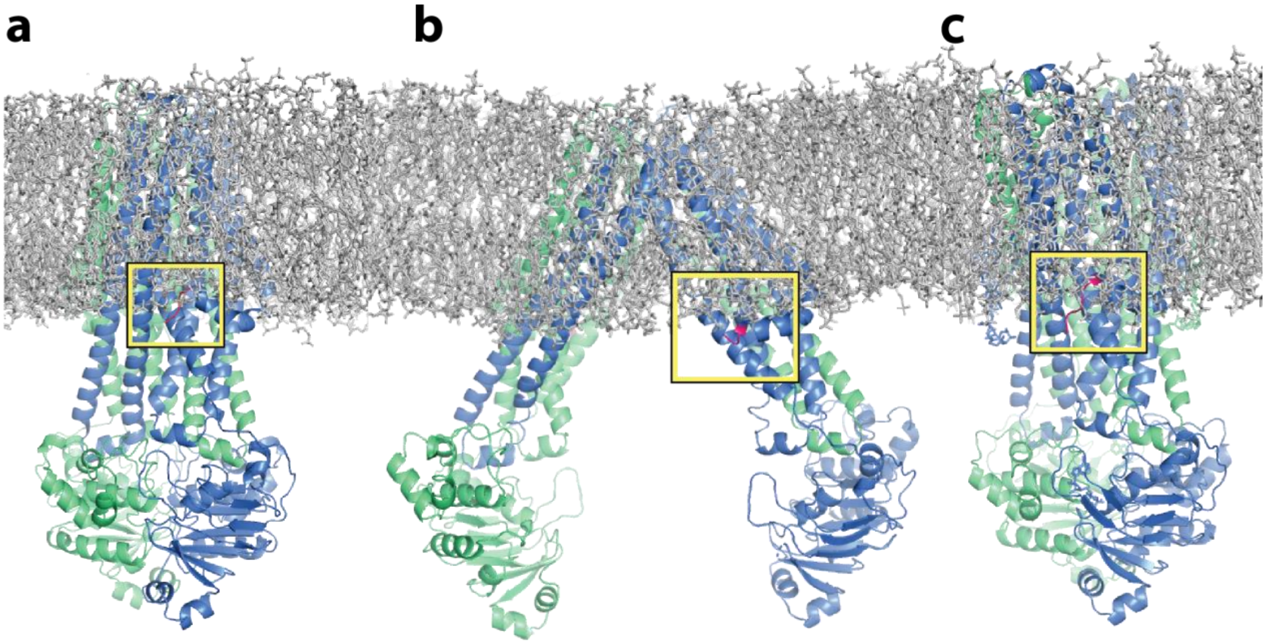

Supplementary Figure 9. N-terminal copper(II) binding site is located in proximity of the bilayer. a-c) Snapshots from molecular dynamics simulation of MsbA structures in 16:0 PC (DPPC) bilayer (PDB 6BPP downloaded from MemProtMD.[7] DPPC lipids are shown in grey sticks, and MsbA in cartoon representation. Part of the N-terminus (first residue in model to residue 9) in colored pink. The yellow box highlights the position of the N-terminus relative to the bilayer. The coordinate files downloaded from MemProtMD are a) 7BCW structure is MSBA in salipro with ADP vanadate, b) open, inward-facing 6BL6 with protein replaced by open, inward-facing MsbA (this report), and c) 3B60 with protein replaced by MSBA trapped with ADP and vanadate.

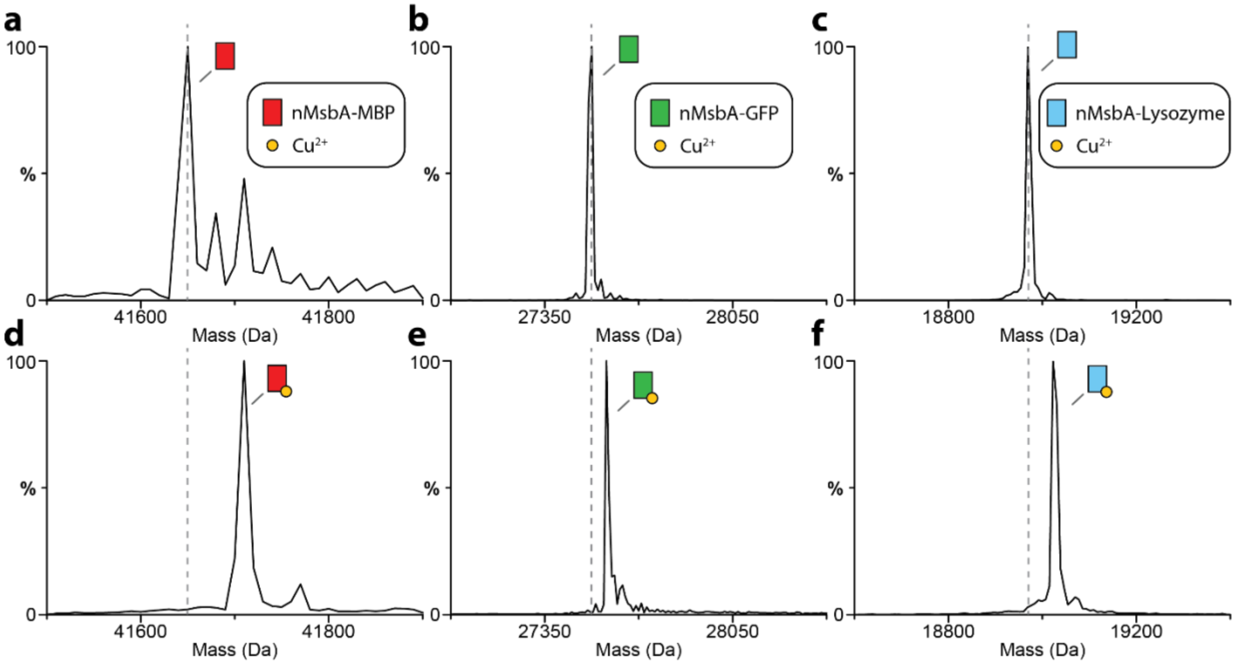

Supplementary Figure 10. Fusion proteins containing a fragment of the N-terminal sequence from MsbA bind copper(II). Shown are the decovoluted mass spectra of a-c) MBP, GFP and T4 lysozyme before addition of copper(II). d-f) MBP, GFP and lysozyme after addition of copper(II).

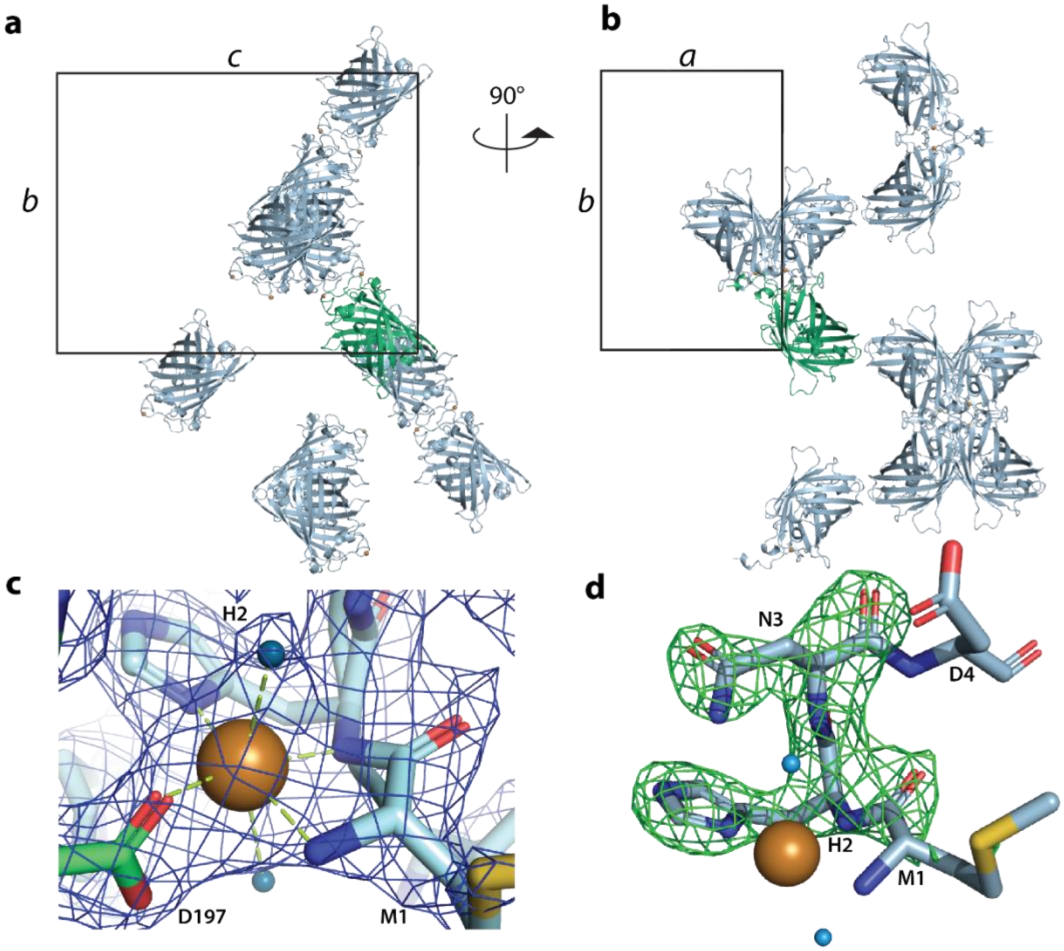

Supplementary Figure 11. Crystal structure of the N-terminal sequence of MsbA fused to GFP and bound to copper(II). a-b) Crystal packing with one molecule (green) in the asymmetric unit cell and symmetry related molecules shown in light blue. c) Coordination of copper(II) within the crystal lattice. D197' from a symmetry related molecule coordinates copper(II). $2F_o–F_c$ electron density map contoured at 1.5 sigma is shown. d) Electron difference density map (F_o-F_c) after refinement with the N-terminus (residues 1-3) omitted and contoured at 5 sigma.

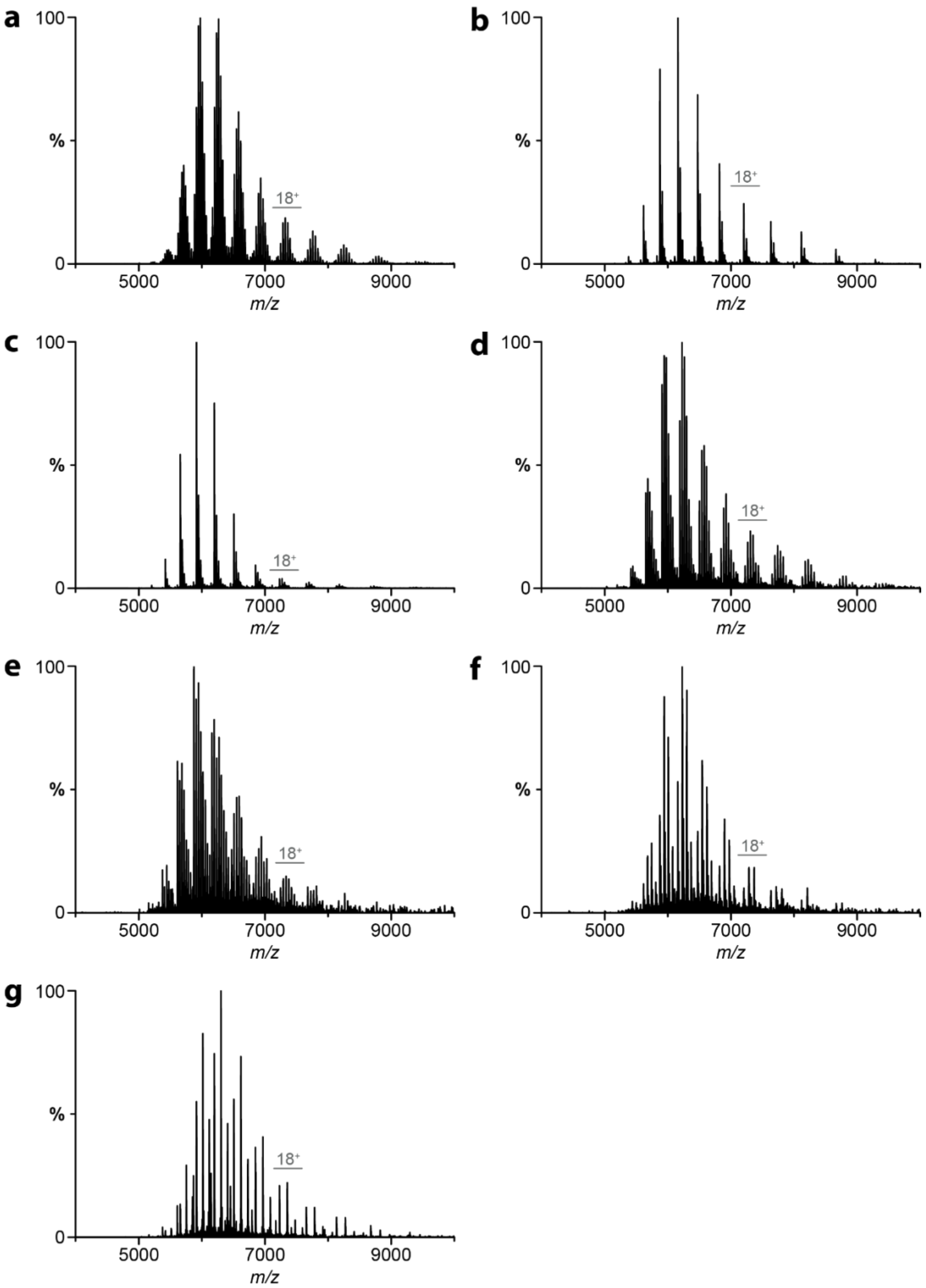

Supplementary Figure 12. Representative native mass spectra for lipids binding to MsbA trapped with ADP and vanadate. MsbA (0.36 μM) was mixed with 6 μM of a) POPA, b) POPC, c) POPE, d) POPG, e) POPS and 1μM of f) TOCDL. g) 0.45 μM MsbA was mixed with 0.7 μM of KDL.

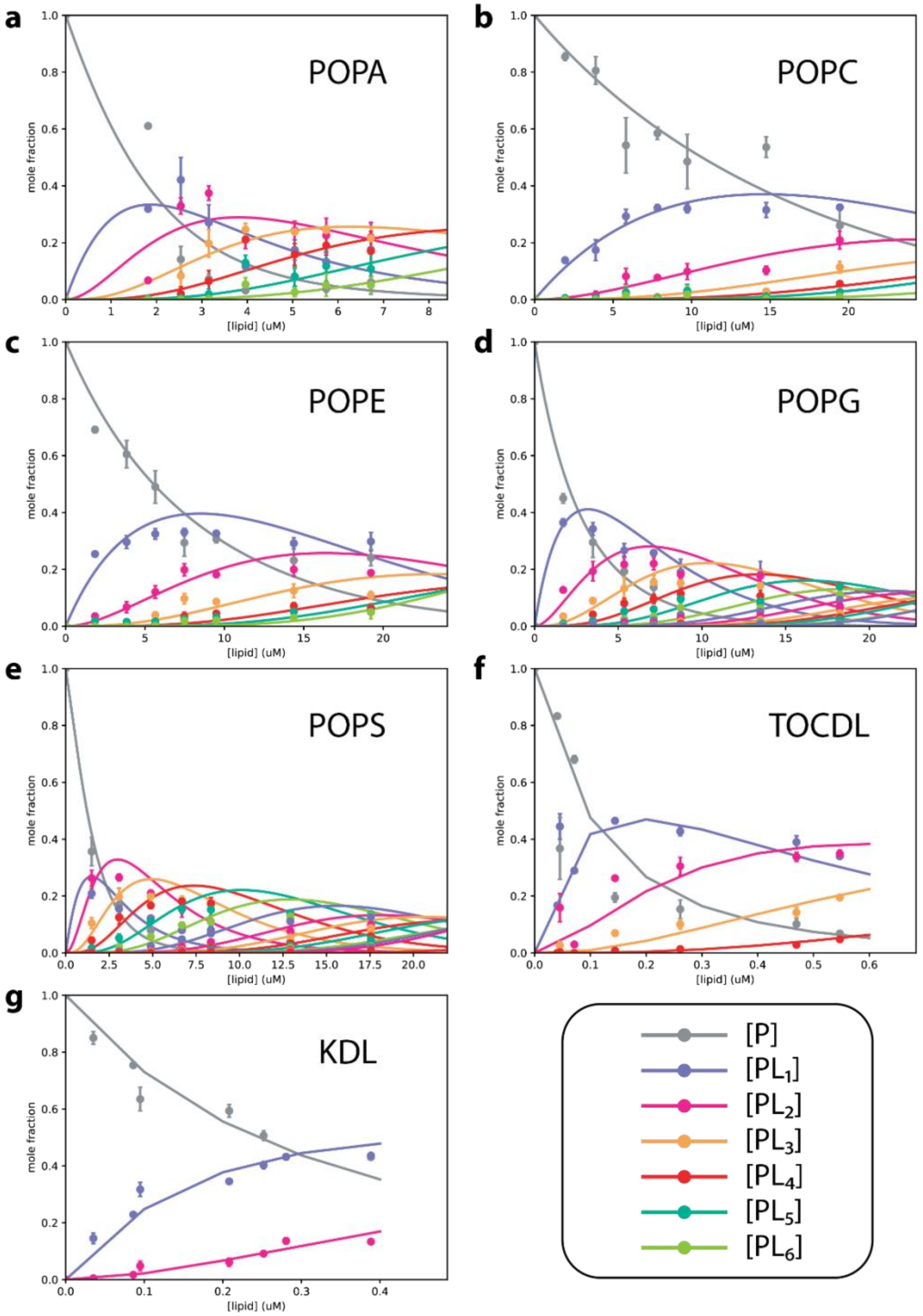

Supplementary Figure 13. Determination of equilibrium dissociation constants (K_D) for lipids binding MsbA trapped with ADP and vanadate. Plots of mole fraction for MsbA bound to different number of a) POPA, b) POPC, c) POPE, d) POPG, e) POPS, f) TOCDL, g) KDL and resulting fit from a sequential lipid-binding model (solid lines). The different lipids are labelled. [P] is the mole fraction of protein and [PL_x] is the mole fraction of protein with x lipids bound. Reported are the mean and standard deviation (*n* = 3 biologically independent samples). Source data are provided as a Source Data file.

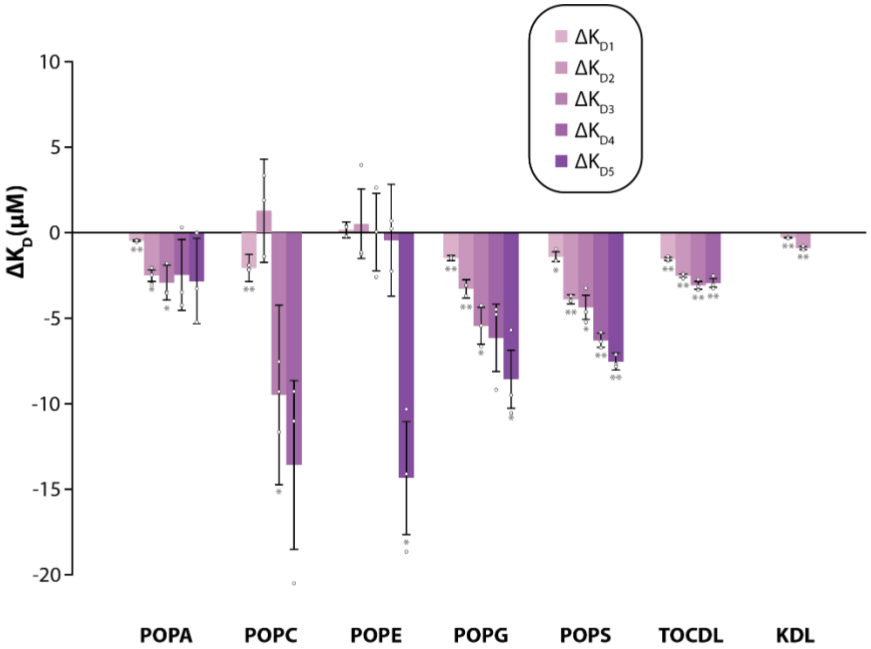

Supplementary Figure 14. The conformation of MsbA influences lipid binding affinity. Reported are the difference in the trapped minus non-trapped K_D values. The asteriks denote student's t-test for MsbA partially versus fully loaded with copper(II). Reported are the mean and standard deviation (n = 3, biological replicates). Two-sided student's t-test (*$p < 0.05$, **$p < 0.01$). p(POPA) = 0.005; 0.012; 0.036. p(POPC) = 0.002; 0.015. p(POPE) = 0.027. p(POPG) = 0.001; 0.004; 0.016; 0.056; 0.028. p(POPS) = 0.025; 0.001; 0.018; 0.002; 0.001. p(TOCDL) = 0.003; 0.001; 0.003; 0.006. p(KDL) = 0.007; 0.005. Source data are provided as a Source Data file.

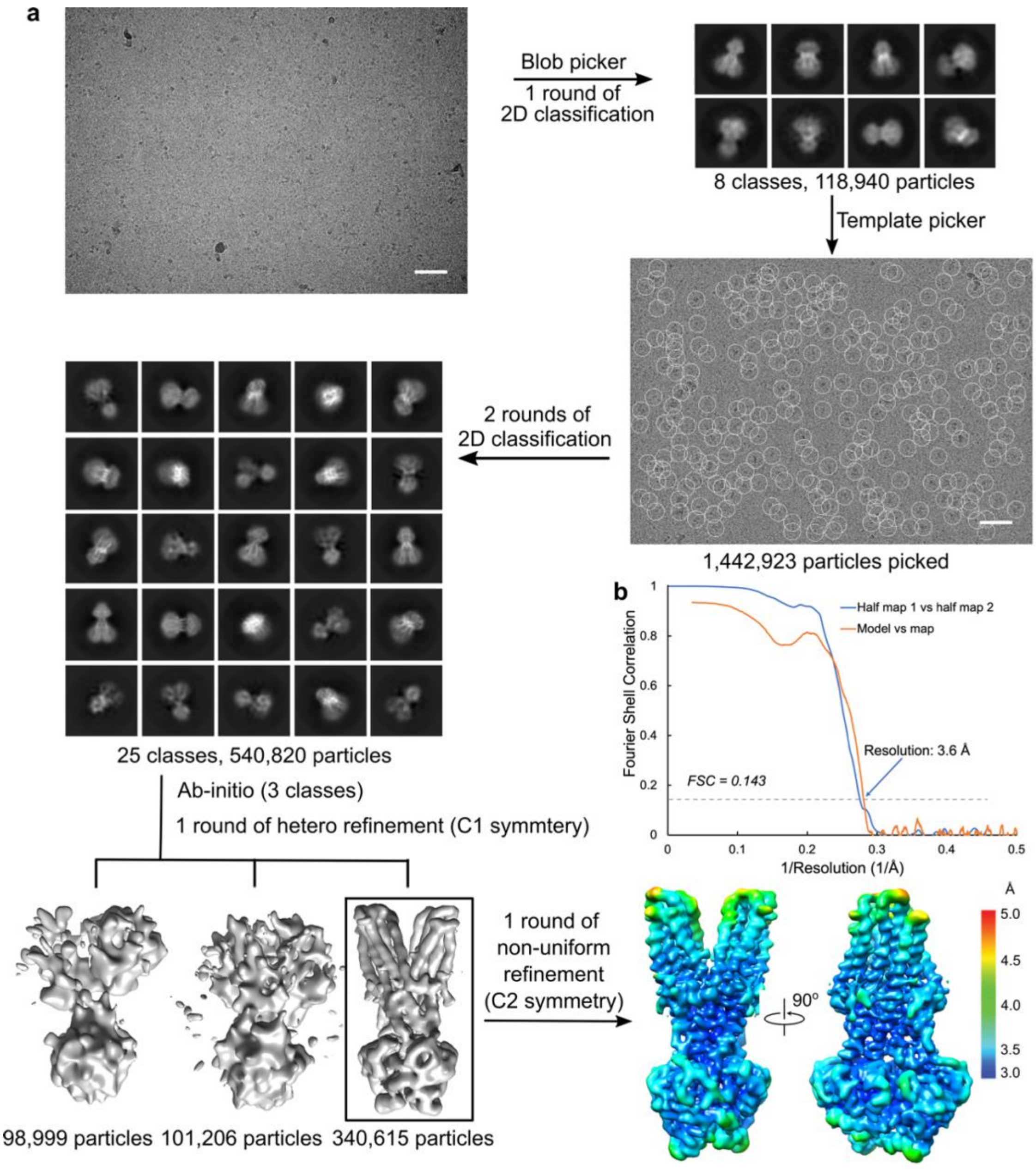

Supplementary Figure 15. Single-particle cryoEM anlaysis for MsbA trapped with vanadate and in complex with KDL. a) The workflow of data processing. Initial motion correction was carried out using MotionCor2.[8] A representative motion-corrected micrograph is shown along with a 50-nm scale bar. The following automatic picking and 2D classification were performed in cryoSPARC.[9] Particle selection was performed using the 2D templates generated by blob picker, followed by a 2D classification. Representative 2D class averages are shown, with the box edge corresponding to 213 Å. After disposing contamination and poorly-aligned 2D classes, the particles were classified by hetero refinement with 3 initial models generated using ab initio reconstructions. A non-uniform refinement was performed on the best class of particles with a C2 symmetry. b) Fourier shell correlation curves of the final reconstruction and model versus map. The resolution of the reconstruction was determined by the FSC=0.143 criterion.

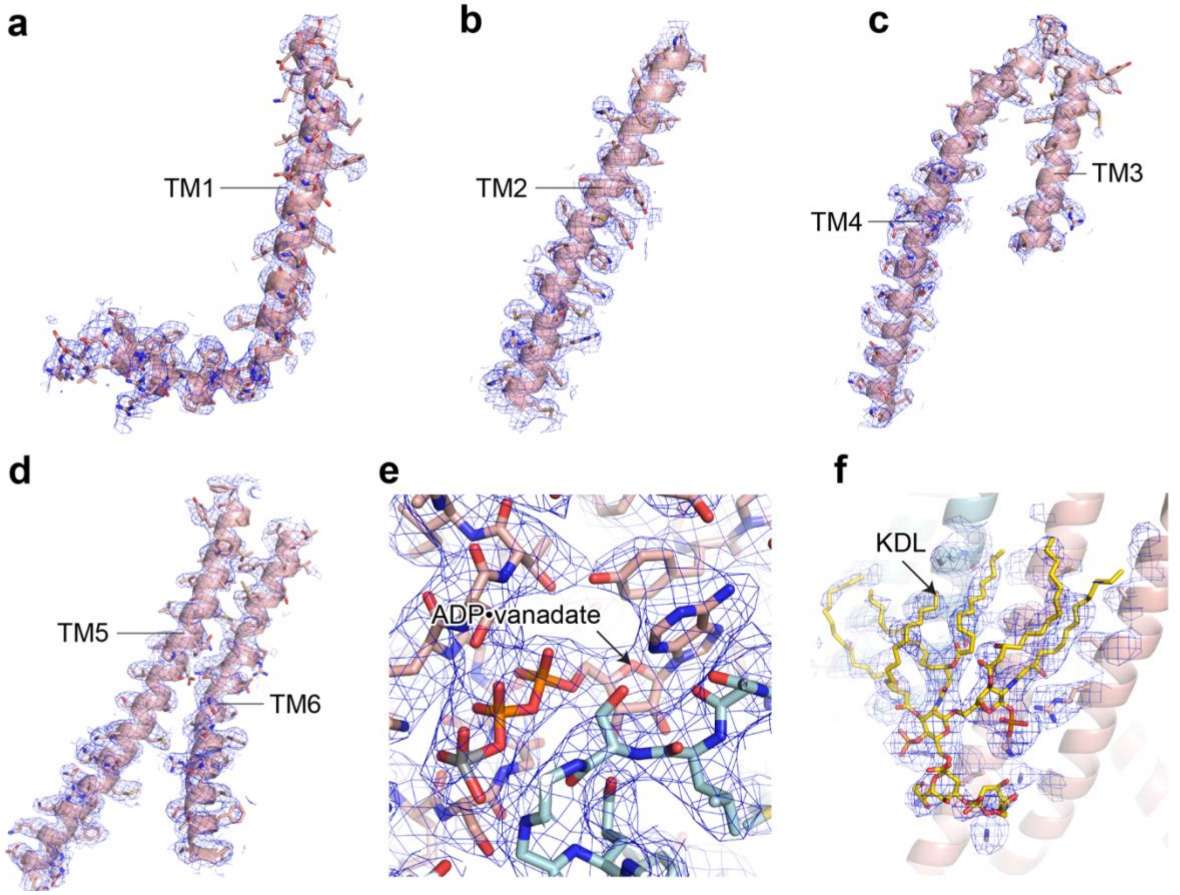

Supplementary Figure 16. Cryo-EM density of the vanadate-trapped MsbA in complex with KDL. a-d) Density (contoured at 4 sigma) and atomic model for six transmembrane helices (TMs). e-f) The bound ADP and vanadate in the nucleotide binding site and exterior bound KDL with density contoured at 5 and 4 sigma, respectively. Figure was prepared using Pymol.[10]

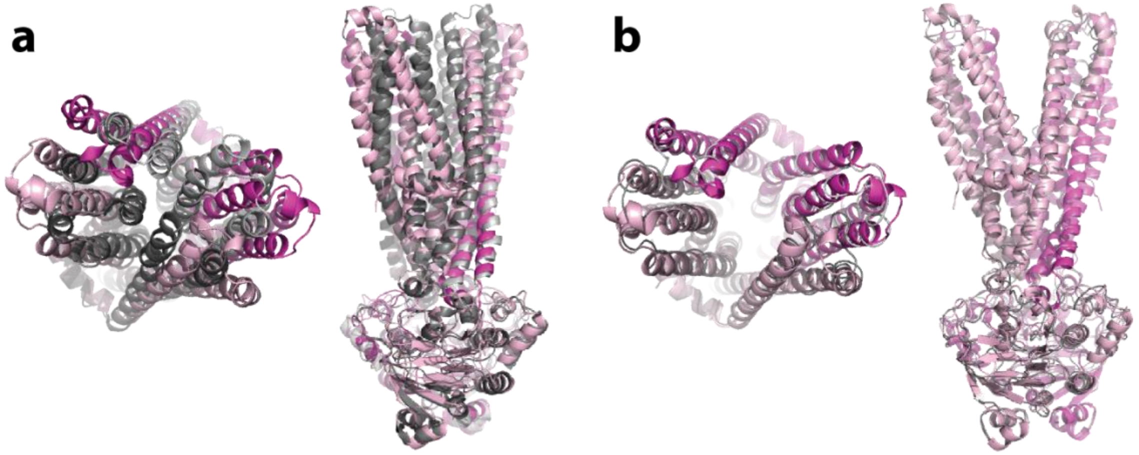

Supplementary Figure 17. Comparison of the vanadate-trapped MsbA structure to other structures. a) Alignment of outward, occluded MsbA structure (PDB 5TTP), shown in pink and magenta, with trapped structure shown in grey. b) Overlay of the MsbA structure with the open, outward facing structure of *S. typhimurium* MsbA bound to AMPPNP, a non-hydrolyzable ATP analog (PDB 3B60). Shown as described for panel a.

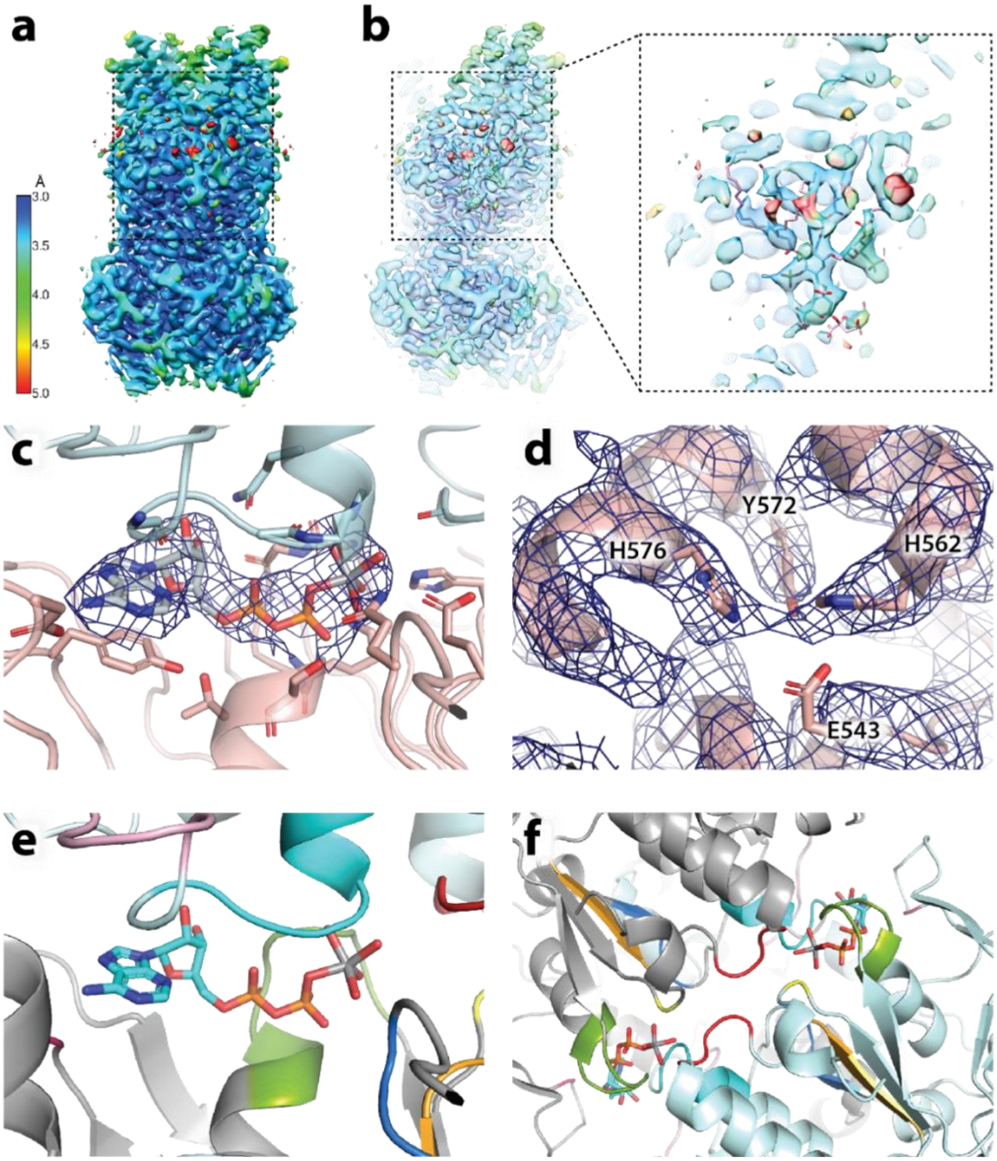

Supplementary Figure 18. CryoEM density and conserved regions of MsbA bound to ADP and vanadate. a) CryoEM map of MsbA bound to ADP, vanadate, and KDL colored by local resolution. b) Map of the exterior KDL binding site contoured at 0.3 in Chimera. c) The same view of the bound nucleotide as shown in Figure 4E with cryoEM density map contoured at 6 rmsd in Coot. d) A previously proposed metal binding site with density map shown as in panel c. Copper is not bound at this location. e-f) Conserved motifs in the ABC transporters. Highlighted are the hydrophobic residue of A-loop (residue 351) in pink, Walker A motif (GxxGxGK(S/T) where x denotes a hydrophobic residue, resides 376-383) or P-loop in chartreuse, Q-loop ($\phi(\phi/Q)Q$ where denotes a hydrophobic residue, residues 421-424), X-loop (residues 472-478) in light pink, C-loop motif (consensus sequence of LSGGQ, residues 481-487) in cyan, Walker B motif (ϕ_4D, residues 501-506) in bright orange, D-loop (consensus sequence of SALD, residues 509-512), and the H-switch histidine (residue 537) in yellow.[11-13] The residue(s) corresponding to the conserved motifs are noted.

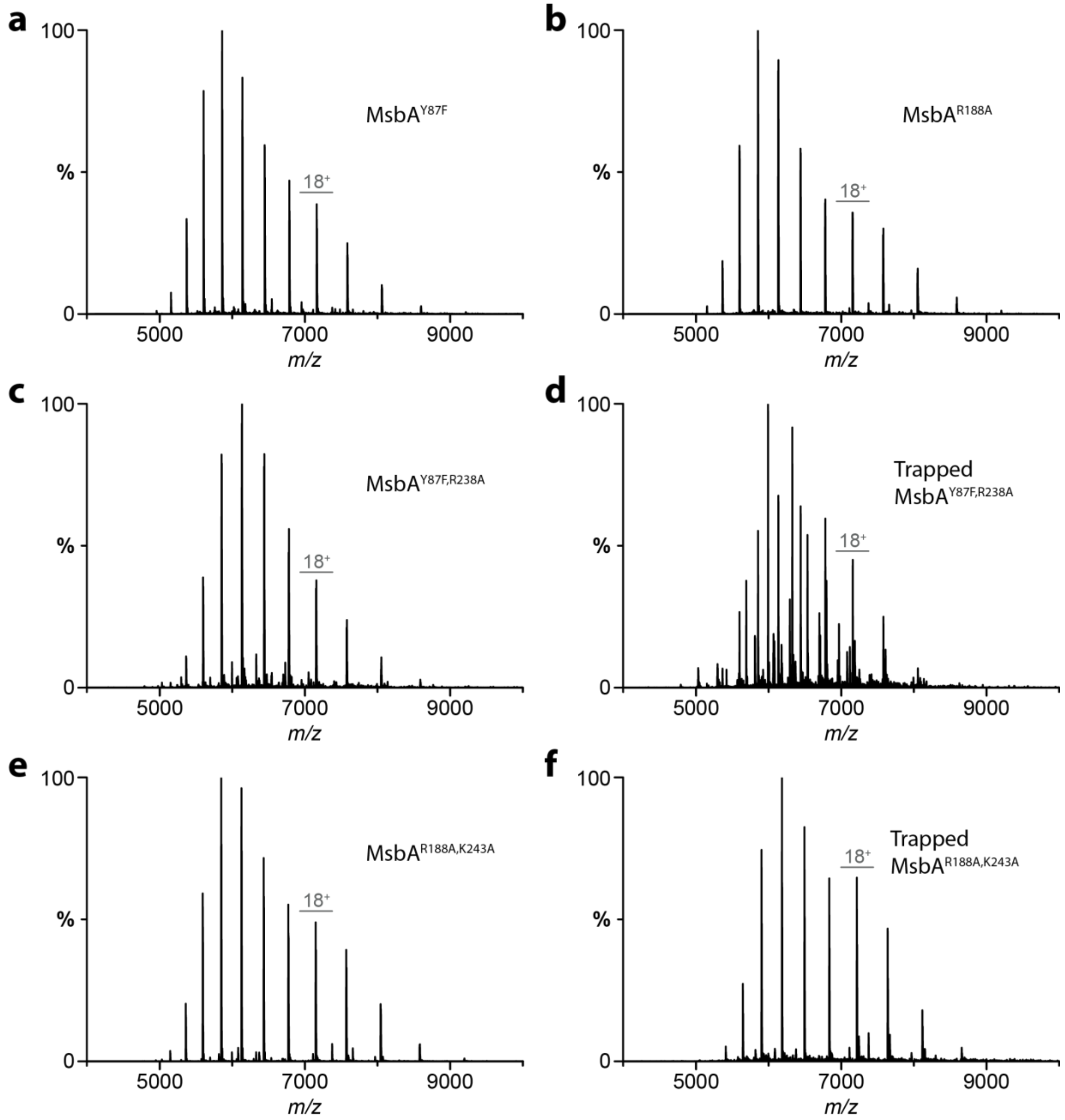

Supplementary Figure 19. Representative native mass spectra of MsbA mutants. a) MsbA^{Y87F} b) MsbA^{R188A} c) MsbA^{Y87F,R238A} d) MsbA^{Y87F,R238A} trapped with ADP and vanadate e) MsbA^{R188A,K243A} f) MsbA^{R188A,K243A} trapped with ADP and vanadate.

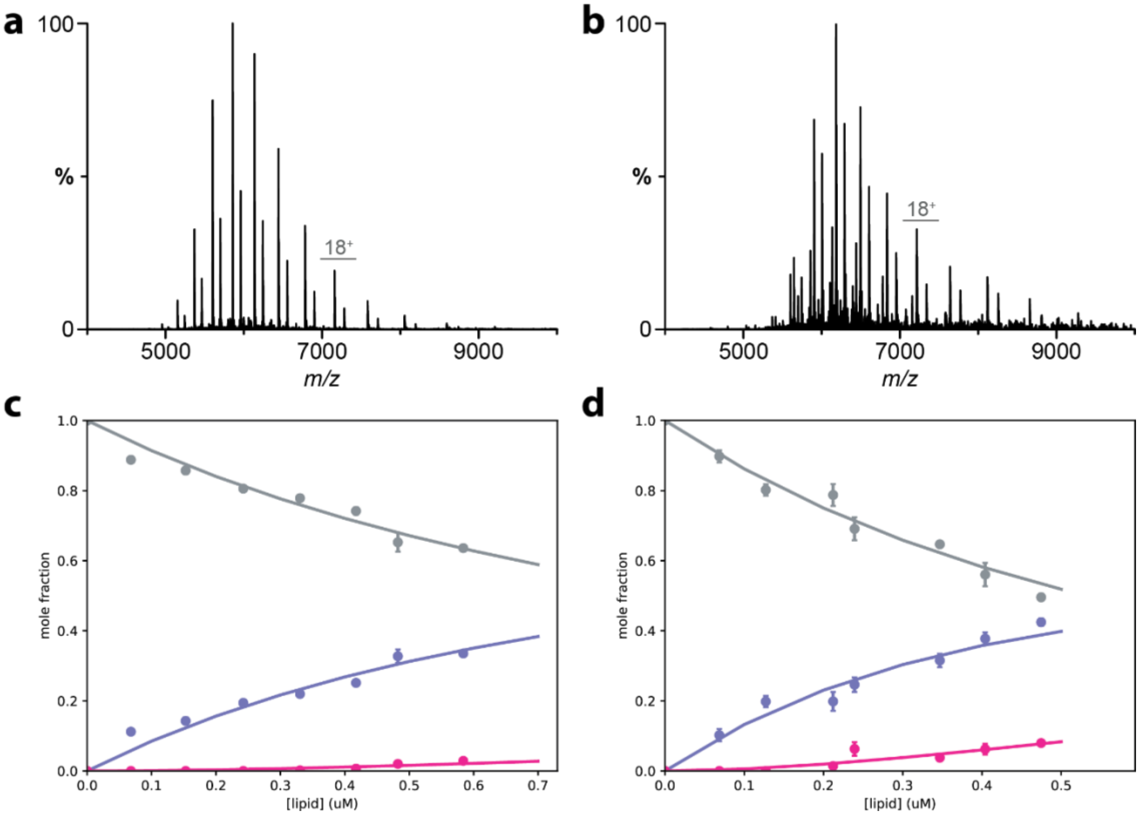

Supplementary Figure 20. Native mass spectra and plot of mole fraction for KDL binding to MsbA^{R188A,R243A}. a) Native mass spectrum of 0.3 µM of MsbA^{R188A,R243A} mixed with 0.7 µM of KDL. b) Native mass spectrum of 0.38 µM of MsbA^{R188A,R243A} trapped with ADP and vanadate and mixed with 0.7 µM of KDL. Mole fraction plots of c) apo and d) vanadate-trapped MsbA^{R188A,R243A} binding to different numbers of KDL. Shown as described in Supplementary Figure 4. Reported are the mean and standard deviation (n = 3 biologically independent samples). Source data are provided as a Source Data file.

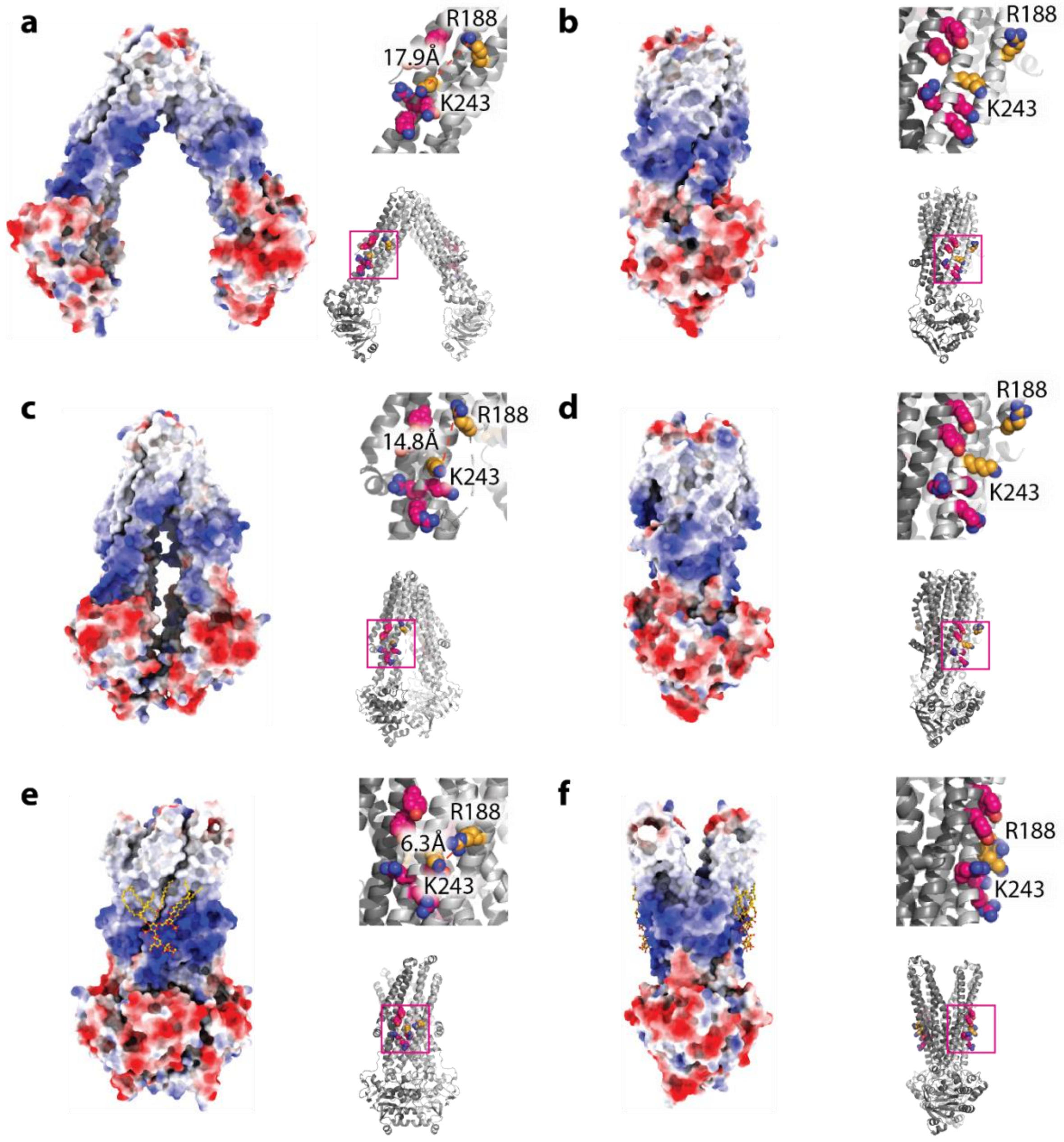

Supplementary Figure 21. Electrostatics of MsbA in different conformations. Shown are 0 and 90° views of a-b) open, inward-facing (this work), c-d) occluded, inward-facing and bound to G907 inhibitor (PDB 6BPL), and e-f) open, outward-facing (this work) conformation. The insets show KDL binding residues in ball and stick representation, and the distance between Cζ of R188 and Nζ of K243 is labeled.

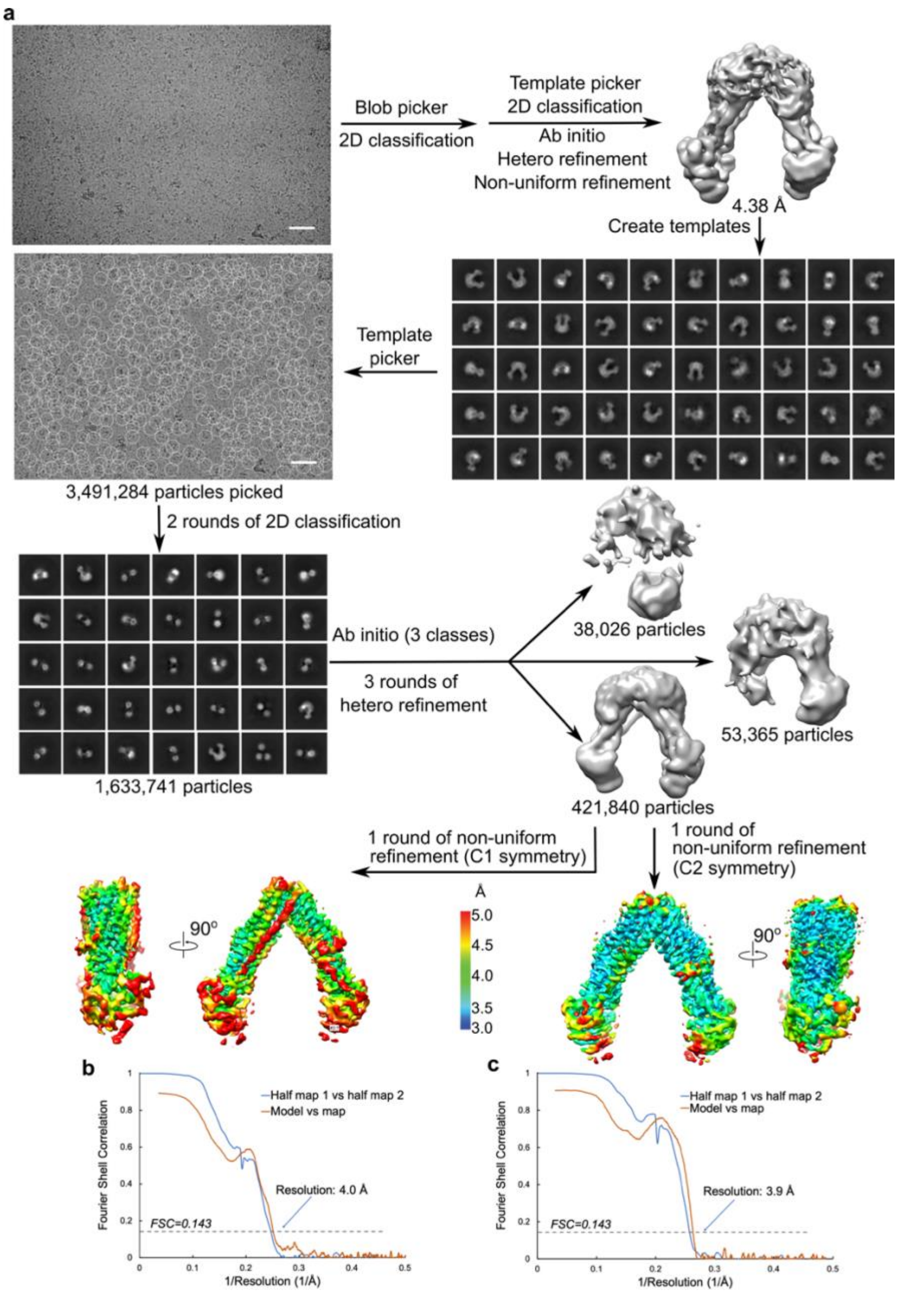

Supplementary Figure 22. Single-particle cryoEM anlaysis for open, inward-facing MsbA. a) The workflow of data processing. The representative micrograph is shown along with a 50-nm scale bar. Particle picking was performed using the 2D templates generated by a reconstructed model from earlier runs. Representative 2D class averages are shown, with the box edge corresponding to 298 Å. After disposing contamination and poorly-aligned 2D classes, the particles were classified by hetero refinement with 3 initial models generated using ab initio reconstructions. A non-uniform refinement was performed on the best class of particles with either C1 or C2 symmetry imposed. b-c) Fourier shell correlation curves of the final reconstruction and model versus map for C1 (b) and C2 (c), respectively. The resolution of the reconstruction was determined by the FSC=0.143 criterion.

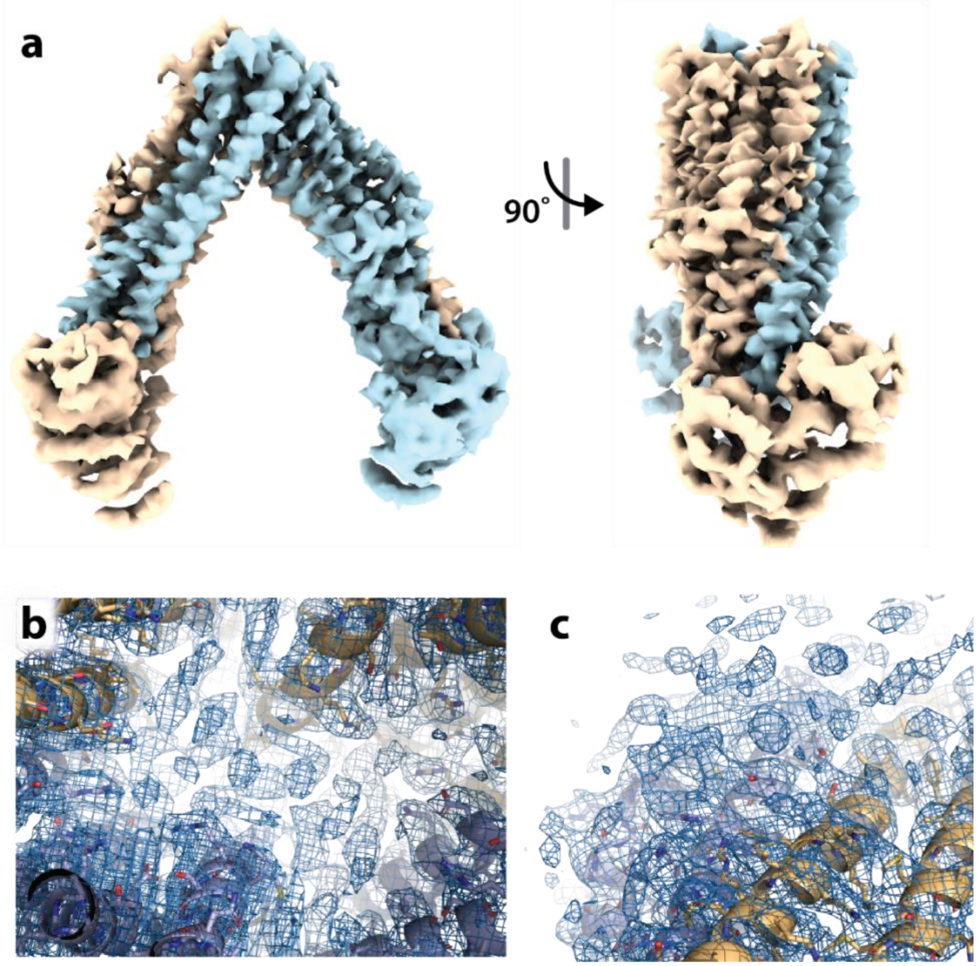

Supplementary Figure 23. CryoEM Structure of apo MsbA in an open, inward facing conformation. a) CryoEM reconstruction (3.8 Å) with the subunits are colored beige and blue. b-c) CryoEM maps showing views b) around residue 87 and c) interior cavity with tube-like density.

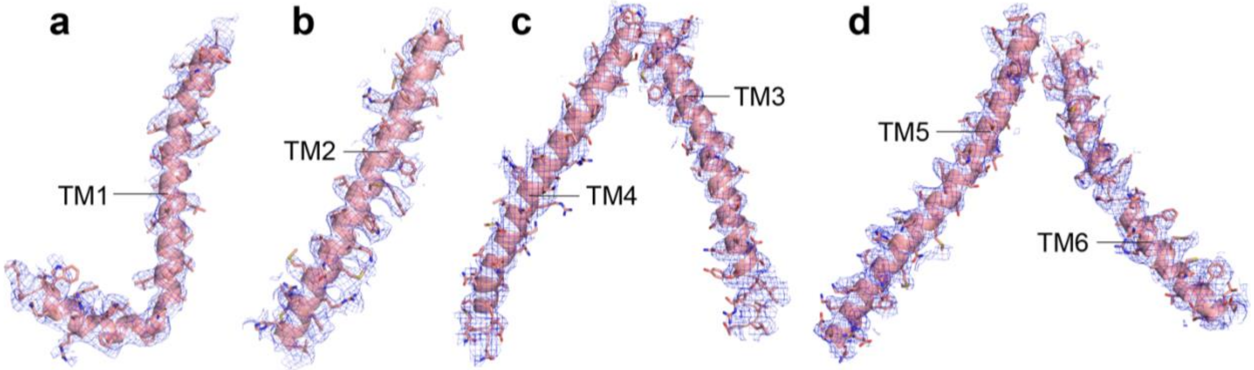

Supplementary Figure 24. Cryo-EM density of the MsbA. a-d) Density (contoured at 8 sigma) and atomic model for six transmembrane helices (TMs). Figure was prepared using Pymol.[10]

Supplementary Tables

Supplementary Table 1. Theoretical and experimental masses of MsbA. Reported are the mean and standard deviation of centroid masses.

	Theoretical (Da)	Experimental (Da)
**$MsbA_2$**	128,947	128,948 ± 7
**$MsbA_2(Cu)_1$**	129,011	129,012 ± 5
**$MsbA_2(Cu)_2$**	129,074	129,079 ± 1
**$MsbA_2(ADP)_2(VO_4)_2$**	130,032	130,005 ± 6
**$MsbA_2(ADP)_2(VO_4)_2(Cu)_1$**	130,095	130,070 ± 4
**$MsbA_2(ADP)_2(VO_4)_2(Cu)_2$**	130,159	130,139 ± 3
**$MsbA_2(Cu)_2(KDL)_1$**	131,309	131,311 ± 5
**$MsbA_2(Cu)_2(KDL)_2$**	133,544	133,548 ± 4

Supplementary Table 2. Operating parameters for ICP-MS analysis of MsbA samples.

Parameters	
**RF Power**	1600 W
**Plasma Ar Flow**	18.0 L min^{-1}
**Auxiliary Ar Flow**	1.20 L min^{-1}
**Nebulizer Ar Flow**	0.98 L min^{-1}
**Sample Introduction System**	Meinhard concentric nebulizer with cyclonic spray chamber
**Operating Frequency**	40 MHz
**Sample Uptake Rate**	1 mL min^{-1}
**Detector Mode**	Analog
**Sampler/Skimmer/Hyper-skimmer Cones**	Ni/Ni/Al
**Scanning Mode**	Peak hopping
**Number of points per peak**	5
**Dwell Time**	50 ms
**Sweeps per Reading**	20
**Isotopes**	^{51}V, ^{60}Ni, ^{63}Cu, ^{66}Zn
**Internal Standards**	^{115}In
**Software**	Syngistix Software, Version 2.4

Supplementary Table 3. Results from ICP-MS analysis of MsbA samples.

Sample	V[ng/mL]	Ni[ng/mL]	Cu[ng/mL]	Zn [ng/mL]
**MsbA**	38	10	1040	7.4
**Vanadate-trapped MsbA**	8338	16	1313	11

Supplementary Table 4. Equilibrium binding constants for lipids binding to MsbA partially loaded with copper(II). K_D was obtained from fitting a sequential lipid binding model to the mole fraction data. Reported are the mean and standard deviation (*n* = 3).

	K_{D1}(μM)	K_{D2}(μM)	K_{D3}(μM)	K_{D4}(μM)	K_{D5}(μM)	K_{D6}(μM)	K_{D7}(μM)	K_{D8}(μM)	K_{D9}(μM)	R^{2*}	X^{2*}
**POPA**	14.7 ± 1.5	23.2 ± 0.6	28.5 ± 2.7	30.8 ± 2.7	32.6 ± 1.9					0.98	0.07
**POPC**	20.4 ± 1.4	39.5 ± 2.0	46.0 ± 4.6	47.0 ± 9.4	34.2 ± 4.6					0.98	0.08
**POPE**	12.4 ± 0.7	23.9 ± 2.4	31.5 ± 3.5	32.5 ± 6.5	33.7 ± 6.9	32.9 ± 4.9	28.0 ± 5.0			0.98	0.08
**POPG**	4.9 ± 0.4	11.1 ± 0.8	17.4 ± 1.7	23.1 ± 0.6	27.1 ± 0.9	28.6 ± 2.0	32.5 ± 3.0			0.95	0.11
**POPS**	3.6 ± 0.2	8.8 ± 0.05	14.7 ± 0.5	20.2 ± 1.3	22.2 ± 1.1	23.1 ± 1.1	25.1 ± 1.0	34.1 ± 1.9	36.7 ± 6.7	0.96	0.10
**TOCDL**	1.9 ± 0.1	4.3 ± 0.7	6.7 ± 0.9	9.4 ± 1.5	12.5 ± 4.0	9.4 ± 1.6				0.99	0.04
**KDL**	0.9 ± 0.1	2.0 ± 0.4	3.5 ± 0.4	5.0 ± 0.3						0.91	0.26

*These values represent the replicates with the poorest fits.

Supplementary Table 5. Equilibrium binding constants for lipids binding to MsbA saturated with copper(II). Reported as described in Table S4.

	K_{D1}(μM)	K_{D2}(μM)	K_{D3}(μM)	K_{D4}(μM)	K_{D5}(μM)	K_{D6}(μM)	K_{D7}(μM)	K_{D8}(μM)	K_{D9}(μM)	R^{2*}	X^{2*}
**POPA**	2.6 ± 0.04	5.7 ± 0.1	8.5 ± 0.3	10.5 ± 0.4	14.0 ± 0.5	16.9 ± 0.3	23.4 ± 3.8			0.99	0.02
**POPC**	17.2 ± 0.6	33.6 ± 1.3	48.5 ± 4.1	53.7 ± 4.9						0.99	0.02
**POPE**	7.4 ± 0.3	18.4 ± 0.7	27.8 ± 0.5	33.1 ± 2.0	39.8 ± 3.2	37.8 ± 4.0				0.99	0.02
**POPG**	4.1 ± 0.1	10.5 ± 0.2	16.2 ± 0.3	20.6 ± 0.7	25.4 ± 1.1	27.6 ± 1.8	31.7 ± 1.6	36.4 ± 1.2	36.4 ± 2.7	1.00	0.01
**POPS**	3.7 ± 0.04	5.7 ± 0.2	9.3 ± 0.6	13.0 ± 0.4	16.8 ± 0.5	19.8 ± 0.5	22.0 ± 0.6	25.4 ± 0.7	27.8 ± 0.1	0.99	0.03
**TOCDL**	1.6 ± 0.08	2.9 ± 0.09	4.1 ± 0.2	5.1 ± 0.2						1.00	0.01
**KDL**	0.6 ± 0.02	2.0 ± 0.09								0.98	0.04

*These values represent the replicates with the poorest fits.

Supplementary Table 6. Summary of X-ray data collection and refinement statistics.

N-term MsbA fusion to GFP in complex with copper(II)		
**Crystal Parameters**		
**Space Group**	$I2_12_12_1$	$I2_12_12_1$
**Unit Cell Dimensions**	*a*=70.61, *b*=108.54, *c*=140.4	*a*=70.62, *b*=108.96, *c*=140.64
**Angles**	α=β=γ=90°	α=β=γ=90°
**Data Collection**		
**Synchrotron (Beamline)**	APS (24-ID-C)	TAMU (R-Axis IV++)
**λ (Å)**	1.378	1.54
**Resolution (Å)**	42.98- 2.15 (2.21- 2.15)	70.32-2.2 (2.28-2.2)
I/σ	12.35 (1.61)	12.17 (2.14)
**Observed Reflections**	60359 (4433)	376860 (27999)
**Unique Reflections**	17601 (1280)	56321 (4109)
**Completeness (%)**	99.3 (98.0)	99.2 (98.6)
**Refinement**		
**R_{work} (%)**	0.19 (0.29)	0.19 (0.34)
**R_{free} (%)**	0.21 (0.30)	0.22 (0.34)
**Number of Protein Atoms**	1968	1955
**Nonprotein Atoms**	26	23
**Number of water molecules**	48	52
**Number of protein residues**	236	236
**RMS (bonds)**	0.008	0.007
**RMS (angles)**	0.91	0.88
**Ramachandran favored (%)**	96.98	97.41
**Ramachandran allowed (%)**	3.02	2.59
**Ramachandran outliers (%)**	0.00	0.00
**Rotamer outliers (%)**	0.96	2.43
**Wilson B-factor**	52.33	57.39
**B factor of protein atoms**	62.70	63.71
**PDB Code**	8DHY	

Supplementary Table 7. Equilibrium binding constants for lipids binding to MsbA trapped with ADP and vanadate. Reported as described in Table S4.

	K_{D1}(μM)	K_{D2}(μM)	K_{D3}(μM)	K_{D4}(μM)	K_{D5}(μM)	K_{D6}(μM)	K_{D7}(μM)	K_{D8}(μM)	K_{D9}(μM)	R^{2*}	X^{2*}
**POPA**	2.2 ± 0.01	3.2 ± 0.3	5.6 ± 10.0	8.0 ± 2.0	11.2 ± 2.4	14.3 ± 2.3				0.84	0.28
**POPC**	15.1 ± 0.5	34.9 ± 2.7	39.0 ± 3.3	40.2 ± 0.3	33.2 ± 3.8					0.95	0.15
**POPE**	7.5 ± 0.4	18.9 ± 1.9	27.8 ± 2.2	32.7 ± 2.6	25.4 ± 0.9	25.2 ± 5.7				0.95	0.13
**POPG**	2.6 ± 0.1	7.2 ± 0.5	10.8 ± 1.0	14.5 ± 1.8	16.8 ± 1.3	21.4 ± 0.5	21.1 ± 0.7	23.4 ± 1.5	27.0 ± 0.6	0.93	0.14
**POPS**	2.3 ± 0.3	1.8 ± 0.2	4.9 ± 0.4	6.7 ± 0.06	9.3 ± 0.2	13.5 ± 0.2	16.2 ± 0.7	20.9 ± 0.6	20.3 ± 1.6	0.93	0.14
**TOCDL**	0.1 ± 0.04	0.4 ± 0.04	1.0 ± 0.04	2.1 ± 0.1						0.86	0.33
**KDL**	0.3 ± 0.02	1.1 ± 0.01								0.97	0.06

*These values represent the replicates with the poorest fits.

Supplementary Table 8. **Statistics of cryo-EM data collection and processing.**

	MsbA trapped with ADP-vanadate and bound to KDL	Open, inward-facing MsbA
**Microscope**	Krios (University of Chicago)	Krios (University of Chicago)
**Magnification**	81,000	81,000
**Voltage (kV)**	300	300
**Spherical aberration (mm)**	2.7	2.7
**Detector**	K3	K3
**Camera mode**	Super resolution counting	Super resolution counting
**Exposure rate (e^{-}/pixel/s)**	15	15
**Total exposure (e^{-}/Å^2)**	50	50
**Defocus range (µm)**	-1.0 to -2.5	-1.0 to -2.5
**Pixel size (Å)**	0.5325 (1.065 physical)	0.5325 (1.065 physical)
**Mode of data collection**	Image shift	Image shift
**Energy filter**	20 eV slit	20 eV slit
**Software for data collection**	EPU	EPU
**Number of micrographs**	4,221	5,530
**Symmetry imposed**	C2	C2
**Box size (pixel)**	200	280
**Initial particle images (no.)**	1,442,923	3,491,284
**Particle images for 3D (no.)**	540,820	1,633,741
**Final particle images (no.)**	340,615	421,840
**Map resolution, unmasked (Å)**	4.0	4.2
**Map resolution, masked (Å)**	3.6	3.9
**B-factor used for sharpening (Å^2)**	213.7	208.6
**EMD accession code**	EMD-27544	EMD-27545

Supplementary Table 9. **Statistics of cryo-EM model refinement and geometry for vanadate-trapped MsbA**

Model	MsbA trapped with ADP-vanadate and bound to KDL		open, inward facing MsbA	
Composition (#)				
Chains	2		2	
Atoms	9314 (Hydrogens: 0)		8912 (Hydrogens: 0)	
Residues	Protein: 1152 Nucleotide: 0		Protein: 1150 Nucleotide: 0	
Water	0		0	
Ligands	KDL: 2		0	
	AOV: 2			
Bonds (RMSD)				
Length (Å) (# > 4σ)	0.004 (0)		0.003 (0)	
Angles (°) (# > 4σ)	0.997 (2)		0.626 (0)	
MolProbity score	1.52		1.66	
Clash score	9.94		8.91	
Ramachandran plot (%)				
Outliers	0.00		0.09	
Allowed	1.13		2.97	
Favored	98.87		96.95	
Rama-Z (Ramachandran plot Z-score, RMSD)				
whole	(N = 1148)	1.55 (0.26)	(N = 1146)	1.76 (0.25)
helix	(N = 728)	1.68 (0.20)	(N = 700)	2.03 (0.20)
sheet	(N = 50)	-0.04 (0.72)	(N = 74)	1.25 (0.61)
loop	(N = 370)	-0.03 (0.35)	(N = 372)	-0.50 (0.32)
Rotamer outliers (%)	0.00		0.00	
Cβ outliers (%)	0.00		0.00	
Peptide plane (%)				
Cis proline/general	0.0/0.0		0.0/0.0	
Twisted proline/general	0.0/0.0		0.0/0.0	
CaBLAM outliers (%)	0.52		1.58	
ADP (B-factors)				
Iso/Aniso (#)	9314/0		8912/0	
min/max/mean				
Protein	21.49/152.72/67.77		0.17/104.70/43.85	
Ligand	53.29/79.81/72.96			
Data				
Box				
Lengths (Å)	75.62, 86.27, 137.39		89.46, 136.32, 136.32	
Angles (°)	90.00, 90.00, 90.00		90.00, 90.00, 90.00	
Supplied Resolution (Å)	3.6		3.9	
Resolution Estimates (Å)	Masked		Masked	
d FSC (half maps; 0.143)	3.6		3.9	
d 99 (full/half1/half2)	3.8/4.5/4.6		4.1/4.6/4.7	
d model	3.8		4.0	
d FSC model (0/0.143/0.5)	3.5/3.6/3.8		3.7/3.8/4.0	
Map min/max/mean	-1.00/1.42/0.04		-1.28/1.91/0.02	
Model vs. Data				
CC (mask)	0.81		0.77	
CC (box)	0.71		0.73	
CC (peaks)	0.67		0.66	
CC (volume)	0.78		0.74	
Mean CC for ligands	0.72		-	

Supplementary References

1 Laganowsky, A., Reading, E., Hopper, J. T. S. & Robinson, C. V. Mass spectrometry of intact membrane protein complexes. *Nature Protocols* **8**, 639-651 (2013). https://doi.org:10.1038/nprot.2013.024

2 Reading, E. *et al.* The Effect of Detergent, Temperature, and Lipid on the Oligomeric State of MscL Constructs: Insights from Mass Spectrometry. *Chem Biol* **22**, 593-603 (2015). https://doi.org:10.1016/j.chembiol.2015.04.016

3 Raetz, C. R., Reynolds, C. M., Trent, M. S. & Bishop, R. E. Lipid A modification systems in gram-negative bacteria. *Annu Rev Biochem* **76**, 295-329 (2007). https://doi.org:10.1146/annurev.biochem.76.010307.145803

4 Mi, W. *et al.* Structural basis of MsbA-mediated lipopolysaccharide transport. *Nature* **549**, 233-237 (2017). https://doi.org:10.1038/nature23649

5 Reading, E. *et al.* The role of the detergent micelle in preserving the structure of membrane proteins in the gas phase. *Angew Chem Int Ed Engl* **54**, 4577-4581 (2015). https://doi.org:10.1002/anie.201411622

6 Laganowsky, A. *et al.* Membrane proteins bind lipids selectively to modulate their structure and function. *Nature* **510**, 172-175 (2014). https://doi.org:10.1038/nature13419

7 Newport, T. D., Sansom, M. S. P. & Stansfeld, P. J. The MemProtMD database: a resource for membrane-embedded protein structures and their lipid interactions. *Nucleic Acids Res* **47**, D390-D397 (2019). https://doi.org:10.1093/nar/gky1047

8 Zheng, S. Q. *et al.* MotionCor2: anisotropic correction of beam-induced motion for improved cryo-electron microscopy. *Nat Methods* **14**, 331-332 (2017). https://doi.org:10.1038/nmeth.4193

9 Punjani, A., Rubinstein, J. L., Fleet, D. J. & Brubaker, M. A. cryoSPARC: algorithms for rapid unsupervised cryo-EM structure determination. *Nat Methods* **14**, 290-296 (2017). https://doi.org:10.1038/nmeth.4169

10 Schrödinger, L. a. W. D. *PyMol v2.4.0*, <http://www.pymol.org/pymol> (2020-05-20).

11 Dawson, R. J. & Locher, K. P. Structure of a bacterial multidrug ABC transporter. *Nature* **443**, 180-185 (2006). https://doi.org:10.1038/nature05155

12 Procko, E., O'Mara, M. L., Bennett, W. F., Tieleman, D. P. & Gaudet, R. The mechanism of ABC transporters: general lessons from structural and functional studies of an antigenic peptide transporter. *FASEB J* **23**, 1287-1302 (2009). https://doi.org:10.1096/fj.08-121855

13 Thomas, C. & Tampe, R. Structural and Mechanistic Principles of ABC Transporters. *Annu Rev Biochem* **89**, 605-636 (2020). https://doi.org:10.1146/annurev-biochem-011520-105201
