# Supplementary material for: Structural basis for lipid and copper regulation of the ABC transporter MsbA
Source: Nat Commun. 2022 Nov 26;13:7291. doi: 10.1038/s41467-022-34905-2 (PMC9701195; doi:10.1038/s41467-022-34905-2)
Supplement: Supplementary file 2 — Reporting Summary [file 41467_2022_34905_MOESM2_ESM.pdf]

nature portfolio

Corresponding author(s): Arthur Laganowsky
Last updated by author(s): Oct 31, 2022

Reporting Summary

Nature Portfolio wishes to improve the reproducibility of the work that we publish. This form provides structure for consistency and transparency in reporting. For further information on Nature Portfolio policies, see our Editorial Policies and the Editorial Policy Checklist.

Statistics

For all statistical analyses, confirm that the following items are present in the figure legend, table legend, main text, or Methods section.

n/a	Confirmed	
☐	☒	The exact sample size (*n*) for each experimental group/condition, given as a discrete number and unit of measurement
☐	☒	A statement on whether measurements were taken from distinct samples or whether the same sample was measured repeatedly
☐	☒	The statistical test(s) used AND whether they are one- or two-sided<br>*Only common tests should be described solely by name; describe more complex techniques in the Methods section.*
☒	☐	A description of all covariates tested
☒	☐	A description of any assumptions or corrections, such as tests of normality and adjustment for multiple comparisons
☐	☒	A full description of the statistical parameters including central tendency (e.g. means) or other basic estimates (e.g. regression coefficient) AND variation (e.g. standard deviation) or associated estimates of uncertainty (e.g. confidence intervals)
☐	☒	For null hypothesis testing, the test statistic (e.g. *F*, *t*, *r*) with confidence intervals, effect sizes, degrees of freedom and *P* value noted<br>*Give P values as exact values whenever suitable.*
☒	☐	For Bayesian analysis, information on the choice of priors and Markov chain Monte Carlo settings
☒	☐	For hierarchical and complex designs, identification of the appropriate level for tests and full reporting of outcomes
☒	☐	Estimates of effect sizes (e.g. Cohen's *d*, Pearson's *r*), indicating how they were calculated

Our web collection on statistics for biologists contains articles on many of the points above.

Software and code

Policy information about availability of computer code

Data collection	Native MS data was acquired on an Orbitrap EMR (Thermo). X-ray crystallography: diffraction data was collected at APS on beamline 24-ID-C. Cryo-EM: ThermoFisher EPU installed on Titan Krios microscope at the Advanced Electron Microscopy Facility at the University of Chicago, Gatan Latitude S installed on Titan Krios microscope at the National Cryo-EM Facility.
Data analysis	Native MS: Unidec, custom python scripts (https://github.com/LaganowskyLab/Laganowsky_Lab_Code), Cryo-EM: MotionCor2 v1.3.2, CTFFIND v4.1.9, RELION v3.1; Structure Visualization: ChimeraX v0.9; Model Building: COOT v0.89, PHENIX v1.20.1.

For manuscripts utilizing custom algorithms or software that are central to the research but not yet described in published literature, software must be made available to editors and reviewers. We strongly encourage code deposition in a community repository (e.g. GitHub). See the Nature Portfolio guidelines for submitting code & software for further information.

Data

Policy information about availability of data

All manuscripts must include a data availability statement. This statement should provide the following information, where applicable:

- Accession codes, unique identifiers, or web links for publicly available datasets
- A description of any restrictions on data availability
- For clinical datasets or third party data, please ensure that the statement adheres to our policy

Cryo-EM maps have been deposited in the Electron Microscopy Data Bank (EMDB, www.ebi.ac.uk/pdbe/emdb/) under the accession codes EMD-27545 and

EMD-27544. Coordinate and structure factors (if appropriate) were deposited in the Protein Data Bank under accession codes 8DHY, 8DMO, and 8DMM. Due to the large file size, raw electron microscopy data are available from the corresponding authors upon request.

Human research participants

Policy information about studies involving human research participants and Sex and Gender in Research.

Reporting on sex and gender	N/A
Population characteristics	N/A
Recruitment	N/A
Ethics oversight	N/A

Note that full information on the approval of the study protocol must also be provided in the manuscript.

Field-specific reporting

Please select the one below that is the best fit for your research. If you are not sure, read the appropriate sections before making your selection.

- [x] Life sciences
- [ ] Behavioural & social sciences
- [ ] Ecological, evolutionary & environmental sciences

For a reference copy of the document with all sections, see nature.com/documents/nr-reporting-summary-flat.pdf

Life sciences study design

All studies must disclose on these points even when the disclosure is negative.

Sample size	The work performed here performed three biological replicates.
Data exclusions	No data was excluded.
Replication	For native MS, three repeated measurements were performed. Replicates were successful for all measurements.
Randomization	Randomization is not relevant as no grouped samples were involved. Replicates were successful.
Blinding	Blinding is not relevant as no grouped samples were involved.

Reporting for specific materials, systems and methods

We require information from authors about some types of materials, experimental systems and methods used in many studies. Here, indicate whether each material, system or method listed is relevant to your study. If you are not sure if a list item applies to your research, read the appropriate section before selecting a response.

Materials & experimental systems

n/a	Involved in the study	
x		Antibodies
x		Eukaryotic cell lines
x		Palaeontology and archaeology
x		Animals and other organisms
x		Clinical data
x		Dual use research of concern

Methods

n/a	Involved in the study	
x		ChIP-seq
x		Flow cytometry
x		MRI-based neuroimaging
